# Tobacco Use and Incidence of Adverse Oral Health Outcomes Among US Adults in the Population Assessment of Tobacco and Health Study

**DOI:** 10.1001/jamanetworkopen.2022.45909

**Published:** 2022-12-09

**Authors:** Marushka L. Silveira, Colm D. Everard, Eva Sharma, Kristin Lauten, Apostolos A. Alexandridis, Kara Duffy, Ethel V. Taylor, Eric A. Tolliver, Carlos Blanco, Wilson M. Compton, Heather L. Kimmel, Timothy Iafolla, Andrew Hyland, Benjamin W. Chaffee

**Affiliations:** 1 National Institute of Dental and Craniofacial Research, National Institutes of Health, Bethesda, Maryland; 2 Kelly Government Solutions, Rockville, Maryland; 3 National Institute on Drug Abuse, National Institutes of Health, Bethesda, Maryland; 4 Westat, Rockville, Maryland; 5 Center for Tobacco Products, US Food and Drug Administration, Silver Spring, Maryland; 6 Department of Health Behavior, Roswell Park Comprehensive Cancer Center, Buffalo, New York; 7 Division of Oral Epidemiology and Dental Public Health, University of California, San Francisco

## Abstract

**Importance:**

Evolving tobacco use patterns, including increasing electronic nicotine delivery systems (ENDS) use, warrant re-examination of the associations between tobacco use and oral health.

**Objective:**

To examine associations between tobacco product use and incidence of adverse oral health outcomes.

**Design, Setting, and Participants:**

This cohort study used nationally representative data from wave (W) 1 to W5 (2013-2019) of the Population Assessment of Tobacco and Health Study. Recruitment used a stratified address-based, area-probability household sample of the noninstitutionalized US civilian population. The W1 cohort included respondents aged 18 years and older without lifetime history of oral health outcomes at W1 or W3, depending on when the outcome was first assessed. Data analysis was performed from October 2021 to September 2022.

**Exposures:**

Current (every day or someday use) established (lifetime use of at least 100 cigarettes or “fairly regular” use of other products) use of cigarettes, ENDS, cigars, pipes, hookah, snus, and smokeless tobacco, excluding snus at W1 to W4.

**Main Outcomes and Measures:**

The primary outcomes were past 12-month self-reported diagnosis of gum disease and precancerous oral lesions (W2-W5) and bone loss around teeth, bleeding after brushing or flossing, loose teeth, and 1 or more teeth removed (W4-W5).

**Results:**

Sample sizes varied across the 6 oral health outcomes (13 149 respondents for the gum disease sample, 14 993 respondents for the precancerous oral lesions sample, 16 312 respondents for the bone loss around teeth sample, 10 286 respondents for the bleeding after brushing or flossing sample, 15 686 respondents for the loose teeth sample, and 12 061 respondents for the 1 or more teeth removed sample). Slightly more than half of adults (52%-54% across the 6 samples) were women, and the majority were of non-Hispanic White race and ethnicity. Cox proportional hazards models were developed with covariates that included time-dependent tobacco use variables mutually adjusted for each other. Cigarette smoking was positively associated with incidence of gum disease diagnosis (adjusted hazard ratio [AHR], 1.33; 95% CI, 1.11-1.60), loose teeth (AHR, 1.35; 95% CI, 1.05-1.75), and 1 or more teeth removed (AHR, 1.43; 95% CI, 1.18-1.74). Cigar smoking was positively associated with incidence of precancerous oral lesions (AHR, 2.18; 95% CI, 1.38-3.43). In addition, hookah smoking was positively associated with incidence of gum disease diagnosis (AHR, 1.78; 95% CI, 1.20-2.63), and ENDS use was positively associated with incidence of bleeding after brushing or flossing (AHR, 1.27; 95% CI, 1.04-1.54). No associations were observed between snus and smokeless tobacco excluding snus and incidence of oral health outcomes.

**Conclusions and Relevance:**

The observed associations of combustible tobacco use with incidence of several adverse oral health outcomes and ENDS use with incidence of bleeding after brushing or flossing highlight the importance of longitudinal studies and emphasize the continued importance of tobacco cessation counseling and resources in clinical practice.

## Key Points

**Question:**

Is the use of tobacco products, especially electronic nicotine delivery systems (ENDS), associated with the incidence of oral health outcomes among US adults?

**Findings:**

This nationally representative cohort study found associations of current combustible tobacco use with the incidence of adverse oral health outcomes and also found an association between current ENDS use and the incidence of bleeding after brushing or flossing.

**Meaning:**

These findings suggest that tobacco product use is associated with the incidence of several adverse oral health outcomes, highlighting the importance of longitudinal studies and emphasizing the continued importance of tobacco cessation counseling and resources in clinical practice.

## Teaser

This cohort study examines associations between tobacco product use and incidence of adverse oral health outcomes among US adults in the Population Assessment of Tobacco and Health (PATH) Study.

## Introduction

Diseases of the teeth and their supporting structures are some of the most prevalent chronic health conditions, affecting approximately 3.5 billion people globally.^1^ These conditions, when untreated, can lead to pain, bone and tooth loss, complex and costly treatments, and reduced quality of life.^2^ Oral diseases have substantial impacts on economic productivity and ability to function at home, school, and work.^3^ Tobacco use is associated with oral disease, and there is sufficient evidence for a causal relationship between cigarette smoking and periodontal disease.^4^ Findings are similar for cigars,^5,6^ pipes,^5,6^ and hookah^7^; however, most of the studies on hookah smoking and periodontal disease have been cross-sectional and conducted in only a few countries, thereby limiting generalizability. Furthermore, use of cigarettes,^4^ cigars,^4^ pipes,^4^ hookah,^7^ and smokeless (chewing) tobacco^4^ is a major cause of oral cavity cancers.

Tobacco-use patterns among adult current smokers have evolved over time, particularly with respect to increasing trends in nondaily cigarette smoking and fewer cigarettes smoked per day.^8^ Yet, cigarettes remain the most commonly used product among adults, followed by electronic nicotine delivery systems (ENDS). These changes in use patterns warrant a re-examination of the associations between combustible and smokeless tobacco use and the incidence of adverse oral health outcomes using recent data. Moreover, literature on oral health effects of ENDS use is limited.^9^ The majority of epidemiological evidence supporting a positive association between ENDS use and adverse oral health comes from cross-sectional studies^10,11,12,13,14^; longitudinal findings have been inconsistent.^15,16^

Understanding health effects of tobacco use, particularly of noncigarette products, is one of the US Food and Drug Administration’s (FDA) tobacco regulatory research priorities.^17^ We examined associations between tobacco use and the incidence of adverse oral health outcomes (ie, gum bleeding, precancerous oral lesions, bone loss around teeth, bleeding after brushing or flossing, loose teeth, and 1 or more teeth removed) across different tobacco products among adults (aged ≥18 years) using data from the Population Assessment of Tobacco and Health (PATH) Study, a unique resource for longitudinal measures on tobacco use and oral health in a US nationally representative sample.^18^

## Methods

### Study Population

Data in this cohort study were from wave (W) 1 (2013-2014), W2 (2014-2015), W3 (2015-2016), W4 (2016-2018), and W5 (2018-2019) of the PATH Study Restricted-Use Files. This study focused on W1 cohort respondents interviewed at all 5 waves who were aged 18 years and older without a lifetime history of each of the 6 oral health outcomes of interest at baseline, defined as W1 or W3 depending on when the oral health outcome was first assessed. Recruitment for the W1 cohort used a stratified address-based, area-probability sampling design that oversampled adult tobacco users, young adults (aged 18-24 years), and African American adults. An in-person screener was used at W1 to randomly select adults from households for participation. Audio computer-assisted self-interviews available in English and Spanish were used to collect information on tobacco-use patterns and health behaviors. Data collection for W2, W3, and W4 followed procedures to interview each respondent approximately 1 year after the prior wave. W5 respondents were interviewed approximately 2 years after W4.

Further details are published elsewhere^19,20,21^ and are described in the PATH Study Restricted-Use Files User Guide.^22^ The PATH Study was conducted by Westat and was approved by the Westat institutional review board. Respondents age 18 years and older provided written informed consent. This report follows the Strengthening the Reporting of Observational Studies in Epidemiology (STROBE) reporting guideline for cohort studies.^23^

### Tobacco Use

Respondents were asked about use of cigarettes, ENDS (“e-cigarettes” at W1 and “electronic nicotine products” in later waves), cigars (ie, traditional cigars, cigarillos, and filtered cigars), pipe tobacco, hookah, smokeless tobacco (including loose snus at W1-W3 and excluding loose snus at W4), and snus (snus pouches at W1-W3 and snus in either loose or pouched form at W4). At each wave, current established cigarette use (yes or no) was defined as having smoked at least 100 cigarettes in one’s lifetime and now smokes every day or some days. For other products, current established use (yes or no) was defined as ever using the product “fairly regularly” and now smokes or uses every day or some days. Detailed definitions for W1 to W4 tobacco use are presented in eTable 1 in Supplement 1.

### Oral Health Outcomes

Self-reported oral health outcomes included gum (periodontal) disease, precancerous oral lesions, bone loss around teeth, bleeding in the mouth after brushing or flossing, loose teeth, and number of teeth removed because of tooth decay or gum disease. These conditions were assessed as lifetime (ever) at baseline and past 12 months at each follow-up wave (see eTable 1 in Supplement 1). Gum disease and precancerous oral lesions were assessed starting in W1, whereas bone loss around teeth, bleeding after brushing or flossing, loose teeth, and number of teeth removed owing to tooth decay or gum disease were assessed starting in W3. Binary (yes or no) variables were created for all W2 to W5 past 12-month outcomes among those without a lifetime history of the specific oral health outcome. The number of teeth removed was dichotomized as 1 or more teeth removed vs none. Participants who were not asked the questions about gum disease, precancerous oral lesions, and bone loss at W4 and W5, because of not having seen a dentist in the past 12 months, were categorized as not having the outcome. Participants who were not asked the loose teeth question at W5 because of having had all their permanent teeth removed owing to tooth decay or gum disease were also categorized as not having the outcome. In addition, the gum disease, precancerous oral lesions, and bone loss around teeth questions were incorrectly skipped by 6 respondents during the first 2 months of W4 data collection and, therefore, were coded as missing.

### Covariates

Time invariant covariates included self-reported baseline age (18-24, 25-34, 35-44, 45-54, 55-64, or ≥65 years), sex (male or female), race and ethnicity (non-Hispanic White, non-Hispanic Black, any other non-Hispanic race including multiracial, or Hispanic), education (less than high school, some high school and no diploma, general education development, high school graduate with a diploma, some college [no degree] or associate degree, or bachelor’s degree or higher), and annual household income (less than $25 000, $25 000-$49 999, $50 000-$74 999, or $75 000 or more). Race and ethnicity were assessed in this study to control for racial and ethnic disparities in tobacco use and oral health outcomes. Missing data on age, sex, race, Hispanic ethnicity, and education were imputed as described in the PATH Study Restricted-Use Files User Guide.^22^

Lifetime history of diabetes (yes or no) and cigarette pack-year computed as the number of cigarette packs smoked per day multiplied by the number of years smoked regularly (categorized as 0, >0 to ≤5, >5 to ≤10, >10 to ≤15, >15 to ≤20, or >20) were included as time invariant covariates based on response at initial wave. Heavy alcohol use (past 30-day consumption of ≥30 drinks among women and ≥60 drinks among men),^16^ past 30-day marijuana use (yes or no), and flossing in the past 7 days (yes or no) for W3 and later waves were included as time-dependent covariates.

### Statistical Analysis

Data analysis was performed from October 2021 to September 2022. Weighted percentages with 95% CIs for all variables were calculated with the survey package in R statistical software version 3.5.1 (R Project for Statistical Computing). Cox proportional hazards models were developed with proc surveyphreg^24,25,26^ in SAS statistical software version 9.4 (SAS Institute). Observations with a positive response for the outcome at any of the waves were coded as 1 = yes regardless of missing data at other waves. Observations with missing data and no positive response for the outcome at other waves were excluded from the analyses because it was unknown whether an event occurred during the wave that was missed. Wave of interview was the time variable. Ties were handled with the Efron method.^25^ Univariate models were developed for time-invariant and time-dependent variables (eTables 2 and 3 in Supplement 1). The proportional hazards assumption was assessed using the log-negative log plot and time-dependent variable methods in SAS using the surveyphreg procedure.^26^

To assess associations between current established tobacco use and incidence of adverse oral health outcomes at the next wave, adjusted hazards ratios (AHRs) and 95% CIs were calculated using Cox proportional hazards models that included current established use of tobacco products as separate variables that were mutually adjusted for each other and time-invariant and time-dependent covariates selected on the basis of literature-supported assumptions about plausible confounders of associations between tobacco use and oral health outcomes^27^ and informed by the univariate models. For the loose teeth outcome, sensitivity analyses excluded 246 respondents who were not asked the question at W5 because of having all their permanent teeth removed. Statistical tests were 2-sided with significance level set at *P* < .05. Full-sample and replicate weights adjusted for the complex sample design and nonresponse. W5 all-waves weights were used, and weighted estimates from W1 to W5 of the W1 cohort represent the resident population of the US aged 12 years and older at the time the data were collected and who were in the civilian noninstitutionalized population at W1.

## Results

Of the 32 320 W1 cohort adult respondents, 18 925 individuals were interviewed at all 5 waves. After excluding 2419 respondents with lifetime history of gum disease and 147 respondents with lifetime history of precancerous oral lesions, analyses were conducted on those not missing data on all variables of interest (13 149 respondents for the gum disease sample and 14 993 respondents for the precancerous oral lesions sample). Of the 28 148 adult respondents at W3, 21 596 were interviewed in W4 and W5. After excluding respondents without lifetime histories of bone loss around teeth (2160 respondents), bleeding after brushing or flossing (9083 respondents), loose teeth (2957 respondents), and 1 or more teeth removed (7324 respondents), analyses were conducted on those with nonmissing data on all variables of interest (16 312 respondents for the bone loss around teeth sample, 10 286 respondents for the bleeding after brushing or flossing sample, 15 686 respondents for the loose teeth sample, and 12 061 respondents for the 1 or more teeth removed sample).

Table 1 shows the baseline characteristics including tobacco product use for the 6 analytic samples. Slightly more than half of adults were women (52%-54% of participants across the 6 samples), and the majority were of non-Hispanic White race and ethnicity. Although prevalence varied across each analytic sample, cigarette smoking was the most commonly used tobacco product followed by cigar use at W1 and ENDS use at W3.

**Table 1.** Tobacco Product Use and Population Characteristics Among Adults in the Analytic Samples for the Gum Disease, Precancerous Oral Lesions, Bone Loss, Bleeding After Brushing or Flossing, Loose Teeth, and 1 or More Teeth Removed Outcomes at Baseline (Wave 1 or Wave 3), Population Assessment of Tobacco and Health Study

Baseline characteristics	Participants, weighted % (95% CI)^a^					
	Wave 1^b^		Wave 3^b^			
	Gum disease sample (n = 13 149)	Precancerous oral lesions sample (n = 14 993)	Bone loss around teeth sample (n = 16 312)	Bleeding after brushing or flossing sample (n = 10 286)	Loose teeth sample (n = 15 686)	≥1 Teeth removed sample (n = 12 061)
Tobacco use, current established use^c^						
Cigarette	16.6 (15.9-17.4)	17.2 (16.6-17.9)	17.0 (16.3-17.7)	18.5 (17.6-19.4)	15.6 (14.9-16.3)	14.2 (13.4-15.1)
Electronic nicotine delivery systems	2.3 (2.0-2.5)	2.4 (2.2-2.6)	3.3 (3.1-3.6)	3.0 (2.7-3.3)	3.2 (2.9-3.5)	3.2 (2.9-3.6)
Cigar	2.9 (2.6-3.1)	2.9 (2.6-3.1)	2.4 (2.2-2.6)	2.5 (2.2-2.8)	2.3 (2.1-2.5)	2.4 (2.1-2.7)
Pipe	0.4 (0.4-0.6)	0.5 (0.4-0.6)	0.3 (0.3-0.5)	0.3 (0.2-0.4)	0.3 (0.3-0.5)	0.3 (0.2-0.4)
Hookah	1.4 (1.2-1.7)	1.3 (1.2-1.5)	1.0 (0.8-1.1)	0.8 (0.7-1.0)	1.0 (0.8-1.1)	1.2 (1.0-1.3)
Smokeless tobacco	2.7 (2.4-3)	2.5 (2.3-2.8)	2.5 (2.3-2.8)	2.3 (2.0-2.7)	2.3 (2.1-2.6)	2.5 (2.2-2.8)
Snus	0.4 (0.3-0.5)	0.4 (0.3-0.5)	0.4 (0.3-0.5)	0.3 (0.2-0.4)	0.3 (0.3-0.5)	0.4 (0.3-0.5)
Sex						
Male	47.2 (46.5-47.9)	47.4 (46.8-47.9)	47.7 (47.1-48.2)	46.0 (44.9-47)	47.0 (46.3-47.6)	47.9 (47.1-48.8)
Female	52.8 (52.1-53.5)	52.7 (52.1-53.2)	52.3 (51.8-52.9)	54.0 (53.0-55.1)	53.0 (52.4-53.7)	52.1 (51.2-52.9)
Age, y						
18-24	14.6 (14.3-15.0)	13.1 (12.8-13.4)	13.6 (13.2-13.9)	11.6 (11.1-12.1)	13.4 (13.1-13.7)	17.9 (17.4-18.4)
25-34	20.8 (20.0-21.7)	19.6 (18.8-20.3)	20.5 (19.7-21.3)	16.3 (15.4-17.2)	19.9 (19.2-20.7)	23.7 (22.7-24.7)
35-44	17.9 (17.1-18.9)	17.7 (16.9-18.5)	17.9 (17.1-18.7)	16.2 (15.2-17.2)	17.7 (16.9-18.6)	19.0 (18.0-20.0)
45-54	17.4 (16.6-18.3)	18.1 (17.4-18.8)	17.1 (16.3-17.9)	16.3 (15.3-17.3)	16.7 (15.9-17.6)	16.6 (15.7-17.6)
55-64	15.0 (14.4-15.7)	16.8 (16.2-17.5)	15.5 (14.8-16.3)	18.3 (17.3-19.3)	16.1 (15.3-17.0)	13.3 (12.5-14.3)
≥65	14.1 (13.3-15.0)	14.7 (14.1-15.4)	15.5 (14.7-16.3)	21.4 (20.5-22.4)	16.1 (15.3-16.9)	9.5 (8.7-10.3)
Race and ethnicity						
Non-Hispanic						
Black	11.2 (10.8-11.7)	11.2 (10.9-11.6)	11.1 (10.7-11.4)	12.1 (11.4-12.8)	10.4 (10.0-10.7)	9.4 (8.9-9.9)
White	65.8 (65.1-66.6)	66.4 (65.8-67.1)	66.0 (65.3-66.7)	64.1 (63.1-65.1)	66.8 (66.2-67.4)	66.6 (65.7-67.5)
Other, including multiracial	7.7 (7.2-8.2)	7.6 (7.2-8.1)	7.8 (7.4-8.2)	8.0 (7.3-8.7)	7.8 (7.4-8.2)	8.6 (7.9-9.3)
Hispanic	15.3 (14.8-15.8)	14.7 (14.3-15.2)	15.2 (14.7-15.7)	15.8 (15.1-16.6)	15.1 (14.6-15.5)	15.4 (14.8-16.1)
Education						
Less than high school or some high school, no diploma	9.8 (9.2-10.4)	9.8 (9.3-10.4)	9.4 (8.9-9.9)	10.5 (9.8-11.3)	8.4 (7.8-9.0)	6.7 (6.1-7.3)
General education development	4.7 (4.3-5.1)	4.8 (4.4-5.2)	5.1 (4.6-5.6)	5.4 (4.8-6.0)	4.6 (4.2-5.1)	3.6 (3.2-4.1)
High school graduate, diploma	23.3 (22.7-23.9)	23.1 (22.6-23.7)	21.9 (21.2-22.6)	23.4 (22.3-24.5)	21.1 (20.5-21.8)	19.1 (18.2-20.0)
Some college (no degree) or associate degree	31.5 (30.9-32.1)	31.6 (31.0-32.1)	32.4 (31.5-33.3)	31.8 (30.7-33.0)	32.5 (31.7-33.4)	32.8 (31.7-33.9)
Bachelor’s degree or more	30.7 (30.1-31.3)	30.7 (30.1-31.2)	31.3 (30.5-32.0)	28.9 (27.8-30.0)	33.3 (32.6-34.1)	37.9 (36.9-38.8)
Annual household income, $						
<25 000	32.3 (31.0-33.6)	32.1 (30.9-33.3)	29.2 (28.3-30.2)	30.8 (29.4-32.2)	26.6 (25.6-27.6)	24.0 (23.1-25.0)
25 000-49 999	22.8 (21.8-23.9)	23.0 (22.0-24.1)	22.4 (21.4-23.5)	23.0 (21.7-24.4)	22.3 (21.3-23.3)	21.0 (19.9-22.1)
50 000-74 999	14.6 (13.7-15.6)	15.1 (14.3-16.0)	16.0 (15.2-16.8)	16.6 (15.6-17.7)	16.6 (15.8-17.5)	16.5 (15.5-17.4)
≥75 000	30.3 (28.8-31.8)	29.8 (28.4-31.3)	32.3 (31.1-33.5)	29.6 (28.1-31.1)	34.4 (33.3-35.6)	38.5 (37.2-39.9)
Flossed in the past 7 d	NA	NA	73.5 (72.3-74.6)	74.0 (72.7-75.3)	75.2 (74.1-76.3)	74.8 (73.7-75.9)
Heavy alcohol use^d^	8.3 (7.6-9.1)	8.5 (7.8-9.2)	8.2 (7.5-8.9)	7.1 (6.4-7.8)	8.0 (7.3-8.8)	8.4 (7.7-9.2)
Past 30-d marijuana use	7.1 (6.5-7.7)	7.1 (6.6-7.7)	10.1 (9.3-10.9)	8.3 (7.6-9.1)	9.5 (8.7-10.2)	10.6 (9.7-11.6)
Cigarette pack-years						
0	48.5 (47.1-49.9)	46.6 (45.2-48.0)	48.3 (46.8-49.8)	47.9 (46.3-49.4)	48.4 (46.8-49.9)	52.3 (50.7-53.8)
>0 to ≤5	28.4 (27.3-29.5)	27.9 (26.9-28.9)	27.5 (26.4-28.6)	24.6 (23.5-25.8)	28.0 (26.8-29.2)	29.8 (28.5-31.0)
>5 to ≤10	5.8 (5.3-6.3)	5.9 (5.4-6.4)	6.2 (5.7-6.7)	5.7 (5.2-6.3)	6.0 (5.5-6.4)	5.3 (4.9-5.8)
>10 to ≤15	3.8 (3.4-4.3)	3.9 (3.5-4.4)	3.9 (3.5-4.3)	4.0 (3.6-4.5)	3.8 (3.4-4.3)	3.3 (2.9-3.8)
>15 to ≤20	3.0 (2.7-3.4)	3.2 (2.9-3.6)	3.0 (2.7-3.4)	3.2 (2.8-3.7)	2.9 (2.6-3.3)	2.6 (2.2-3.0)
>20	10.5 (9.7-11.3)	12.4 (11.7-13.2)	11.2 (10.5-11.9)	14.6 (13.6-15.6)	10.9 (10.3-11.6)	6.8 (6.1-7.5)
Lifetime history of diabetes^e^	12.6 (11.8-13.4)	14.0 (13.2-14.8)	16.7 (15.9-17.5)	19.0 (17.7-20.3)	16.9 (16.0-17.8)	13.1 (12.1-14.1)

Abbreviation: NA, not applicable.

^a^ Percentages (95% CI) are weighted.

^b^ Restricted to participants with data on all variables in the model for the specific oral health outcome.

^c^ Current established use (yes or no) for cigarettes was defined as having smoked at least 100 cigarettes in one’s lifetime and now smokes every day or some days. For other products, current established use (yes or no) was defined as ever using the product “fairly regularly” and now smokes or uses every day or some days.

^d^ Heavy alcohol use was defined as consumption of 30 or more drinks among women and 60 or more drinks among men in the past 30 days.

^e^ Reference is yes vs no.

Unadjusted associations between current established tobacco product use, covariates, and oral health outcomes are presented in eTable 2 and eTable 3 in Supplement 1. Table 2 summarizes the sample sizes, number of events, and censored values for each outcome in the Cox proportional hazards models presented in Table 3 and Table 4. For gum disease and precancerous oral lesions, follow-up time was 4 waves (approximately 5 years). For bone loss around teeth, bleeding after brushing or flossing, loose teeth, and 1 or more teeth removed was 2 waves (approximately 3 years).

**Table 2.** Summary of the Unweighted and Weighted Number of Incident Adverse Oral Health Events Among Adults Over the Past 12 Months and Censored Values for the Cox Proportional Hazards Models With Covariates, Population Assessment of Tobacco and Health Study

Incidence of past 12-mo oral health outcomes	Waves assessed	Unweighted			Weighted		
		Participants, No.	Events, No.	Censored, No. (%)	Participants, thousands	Events, thousands	Censored, thousands, No. (%)
Gum disease^a^	2-5	13 149	1166	11 983 (91.13)	154 510	12 668	141 840 (91.80)
Precancerous oral lesions^b^	2-5	14 993	205	14 788 (98.63)	177 020	1872	175 150 (98.94)
Bone loss around teeth^c^	4-5	16 312	794	15 518 (95.13)	173 190	9300	163 890 (94.63)
Bleeding after brushing or flossing^d^	4-5	10 286	2285	8001 (77.79)	113 770	20 020	93 749 (82.40)
Loose teeth^e^	4-5	15 686	872	14 814 (94.44)	170 990	8114	162 870 (95.25)
≥1 Teeth removed^f^	4-5	12 061	1264	10 797 (89.52)	121 640	12 176	109 460 (89.99)

^a^ Gum disease was defined according to a positive response to the question whether participants were told by a dentist, hygienist, or other health professional that they had gum disease in the past 12 months regardless of missing data at any of the waves. Participants who responded no to this question at waves 2 and 3 and who either responded no to this question or were not asked the question because of not having seen a dentist in the past 12 months at waves 4 and 5 are categorized as not having the outcome.

^b^ Precancerous oral lesions were defined according to a positive response to the question whether participants were told by a dentist, hygienist, or other health professional that they had precancerous oral lesions in the past 12 months regardless of missing data at any of the waves. Participants who responded no to this question at waves 2 and 3 and who either responded no to this question or were not asked the question because of not having seen a dentist in the past 12 months at waves 4 and 5 are categorized as not having the outcome.

^c^ Bone loss around teeth was defined according to a positive response to the question whether participants were told by a dentist, hygienist, or other health professional that they had lost bone around their teeth in the past 12 months regardless of missing data at any of the waves. Participants who either responded no to this question or were not asked this question because of not having seen a dentist in the past 12 months at wave 4 and who either responded no to this question or were not asked this question because of not having seen a dentist in the past 12 months or did not know or refused to report whether they have seen a dentist in the past 12 months at wave 5 were categorized as not having the outcome.

^d^ Bleeding after brushing or flossing was defined according to a positive response to the question whether participants had observed any bleeding after brushing or flossing, or due to other conditions in their mouth in the past 12 months regardless of missing data at any of the waves. Participants who responded no to this question at waves 4 and 5 were categorized as not having the outcome.

^e^ Loose teeth was defined according to a positive response to the question whether participants had any teeth become loose on their own, without an injury in the past 12 months regardless of missing data at any of the waves. Participants who responded no to this question at wave 4 and who either responded no to this question or were not asked this question because of having had all their permanent teeth removed at wave 5 were categorized as not having the outcome.

^f^ One or more teeth removed was defined according to greater than 0 teeth removed to the question regarding how many of participants’ permanent teeth had been removed because of tooth decay or gum disease in the past 12 months regardless of missing data at any of the waves. Those who responded 0 to this question at waves 4 and 5 were categorized as not having the outcome.

**Table 3.** Cox Proportional Hazards Models With Covariates Assessing Associations of Time-Dependent Tobacco Product Use With Incidence of Gum Disease and Precancerous Oral Lesions, Population Assessment of Tobacco and Health Study

Time-dependent current established tobacco use from waves 1 to 4^a^	Incidence of oral health outcomes from waves 2 to 5			
	Gum disease^b^		Precancerous oral lesions^c^	
	AHR (95% CI)^d^	*P* value	AHR (95% CI)^d^	*P* value
Cigarette	1.33 (1.11-1.60)	.002	1.47 (0.87-2.48)	.15
Electronic nicotine delivery systems	1.15 (0.89-1.47)	.28	0.56 (0.26-1.20)	.14
Cigar	1.14 (0.83-1.57)	.42	2.18 (1.38-3.43)	.001
Pipe	0.85 (0.38-1.86)	.67	0.77 (0.22-2.67)	.68
Hookah	1.78 (1.20-2.63)	.005	2.70 (0.91-7.99)	.07
Smokeless tobacco	0.88 (0.61-1.28)	.50	1.66 (0.90-3.07)	.10
Snus	0.90 (0.26-3.10)	.87	0.73 (0.16-3.41)	.68

Abbreviation: AHR, adjusted hazard ratio.

^a^ Current established use (yes or no) for cigarettes was defined as having smoked at least 100 cigarettes in one’s lifetime and now smokes every day or some days. For other products, current established use (yes or no) was defined as ever using the product “fairly regularly” and now smokes or uses every day or some days. Reference is yes vs no.

^b^ Gum disease was defined according to a positive response to the question whether participants were told by a dentist, hygienist, or other health professional that they had gum disease in the past 12 months regardless of missing data at any of the waves. Participants who responded no to this question at waves 2 and 3 and who either responded no to this question or were not asked the question because of not having seen a dentist in the past 12 months at waves 4 and 5 are categorized as not having the outcome.

^c^ Precancerous oral lesions were defined according to a positive response to the question whether participants were told by a dentist, hygienist, or other health professional that they had precancerous oral lesions in the past 12 months regardless of missing data at any of the waves. Participants who responded no to this question at waves 2 and 3 and who either responded no to this question or were not asked the question because of not having seen a dentist in the past 12 months at waves 4 and 5 are categorized as not having the outcome.

^d^ AHRs and 95% CIs are from survival analysis models adjusting for time-invariant wave 1 (age, sex, race and ethnicity, educational attainment, annual household income, cigarette pack-years, and lifetime history of diabetes) and time-dependent waves 1 to 4 (heavy alcohol use and past 30-day marijuana use) covariates. Models are weighted using the wave 5 all-waves weights for the wave 1 cohort.

**Table 4.** Cox Proportional Hazards Models With Covariates Assessing Associations of Time-Dependent Tobacco Product Use With Incidence of Bone Loss Around Teeth, Bleeding After Brushing or Flossing, Loose Teeth, and 1 or More Teeth Removed, Population Assessment of Tobacco and Health Study

Time-dependent, current established tobacco use from waves 3 to 4^a^	Incidence of oral health outcomes from waves 4 to 5							
	Bone loss around teeth^b^		Bleeding after brushing or flossing^c^		Loose teeth^d^		One or more teeth removed^e^	
	AHR (95% CI)^f^	*P* value	AHR (95% CI)^f^	*P* value	AHR (95% CI)^f^	*P* value	AHR (95% CI)^f^	*P* value
Cigarette	0.99 (0.77-1.27)	.91	0.94 (0.81-1.10)	.43	1.35 (1.05-1.75)	.02	1.43 (1.18-1.74)	<.001
Electronic nicotine delivery systems	0.95 (0.69-1.31)	.75	1.27 (1.04-1.54)	.02	1.01 (0.75-1.35)	.97	1.03 (0.80-1.33)	.81
Cigar	0.85 (0.57-1.25)	.40	1.04 (0.82-1.30)	.76	1.41 (0.99-1.99)	.06	1.26 (0.96-1.64)	.09
Pipe	1.41 (0.57-3.47)	.46	0.87 (0.35-2.20)	.77	1.36 (0.46-4.05)	.58	1.69 (0.84-3.38)	.14
Hookah	1.04 (0.51-2.12)	.92	0.98 (0.70-1.36)	.88	0.69 (0.36-1.33)	.26	0.85 (0.48-1.50)	.56
Smokeless tobacco	0.72 (0.33-1.57)	.41	1.02 (0.81-1.28)	.89	0.86 (0.54-1.38)	.53	1.13 (0.79-1.63)	.49
Snus	0.95 (0.23-4.00)	.94	0.77 (0.32-1.85)	.56	1.55 (0.68-3.56)	.29	1.26 (0.69-2.31)	.45

Abbreviation: AHR, adjusted hazard ratio.

^a^ Current established use (yes or no) for cigarettes was defined as having smoked at least 100 cigarettes in one’s lifetime and now smokes every day or some days. For other products, current established use (yes or no) was defined as ever using the product “fairly regularly” and now smokes or uses every day or some days. Reference is yes vs no.

^b^ Bone loss around teeth was defined according to a positive response to the question whether participants were told by a dentist, hygienist, or other health professional that they had lost bone around their teeth in the past 12 months regardless of missing data at any of the waves. Participants who either responded no to this question or were not asked this question because of not having seen a dentist in the past 12 months at wave 4 and who either responded no to this question or were not asked this question because of not having seen a dentist in the past 12 months or did not know or refused to report whether they have seen a dentist in the past 12 months at wave 5 were categorized as not having the outcome.

^c^ Bleeding after brushing or flossing was defined according to a positive response to the question whether participants had observed any bleeding after brushing or flossing, or due to other conditions in their mouth in the past 12 months regardless of missing data at any of the waves. Participants who responded no to this question at waves 4 and 5 were categorized as not having the outcome.

^d^ Loose teeth was defined according to a positive response to the question whether participants had any teeth become loose on their own, without an injury in the past 12 months regardless of missing data at any of the waves. Participants who responded no to this question at wave 4 and who either responded no to this question or were not asked this question because of having had all their permanent teeth removed at wave 5 were categorized as not having the outcome.

^e^ One or more teeth removed was defined according to greater than 0 teeth removed to the question regarding how many of participants’ permanent teeth had been removed because of tooth decay or gum disease in the past 12 months regardless of missing data at any of the waves. Those who responded 0 to this question at waves 4 and 5 were categorized as not having the outcome.

^f^ AHRs and 95% CIs are from survival analysis models adjusting for time-invariant wave 3 (age, sex, race and ethnicity, educational attainment, annual household income, cigarette pack-years, and lifetime history of diabetes) and time-dependent waves 3 and 4 (flossing during the past 7 days, heavy alcohol use, and past 30-day marijuana use) covariates. Models are weighted using the wave 5 all-waves weights for the wave 1 cohort.

Table 3 presents the AHRs for associations between time-dependent current established tobacco product use from W1 to W4 and incidence of oral health outcomes from W2 to W5. AHRs for all covariates are presented in eTable 4 in Supplement 1. Cigarette (AHR, 1.33; 95% CI, 1.11-1.60) and hookah (AHR, 1.78; 95% CI, 1.20-2.63) smoking were each positively associated with incidence of gum disease diagnosis. Smoking any cigars (AHR, 2.18; 95% CI, 1.38-3.43) was positively associated with incidence of precancerous oral lesions. Snus (ie, snus pouches only at W1-W3 and any snus at W4) and smokeless tobacco (excluding snus) were not significantly associated with incidence of gum disease diagnosis or precancerous oral lesions.

Table 4 presents the AHRs for associations between time-dependent current established tobacco product use from W3 to W4 and incidence of oral health outcomes from W4 to W5. AHRs for all covariates are presented in eTable 5 in Supplement 1. Cigarette smoking was positively associated with incidence of loose teeth (AHR, 1.35; 95% CI, 1.05-1.75) and 1 or more teeth removed (AHR, 1.43; 95% CI, 1.18-1.74). In addition, ENDS use was positively associated with the incidence of bleeding after brushing or flossing (AHR, 1.27; 95% CI, 1.04-1.54). Findings were similar for all tobacco products when respondents who were not asked the loose teeth question at W5 were excluded (data not shown). Snus (ie, snus pouches only at W1-W3 and any snus at W4) and smokeless tobacco (excluding snus) use were not associated with any of the oral health outcomes.

All time-invariant variables in our models satisfied the proportional hazards assumption with *P* values from adjusted Wald *F* tests being greater than .05, except for age (AHR, 0.94; 95% CI, 0.90-0.99; *P* = .02) and lifetime history of diabetes (AHR, 0.76; 95% CI, 0.62-0.93; *P* = .01) in the gum disease model, race or ethnicity (AHR, 0.90; 95% CI, 0.82-0.99; *P* = .03) in the bleeding after brushing or flossing model, and cigarette pack-years (AHR, 1.10; 95% CI, 1.01-1.20; *P* = .03) in the any teeth removed model; including the interaction with time terms for these variables, within the specified models, did not result in any substantive changes in the results presented (data not shown).

## Discussion

This nationally representative cohort study of US adults confirms associations between tobacco use and incidence of oral health outcomes among those without a lifetime history of the oral health outcome. Associations were robust to adjustment for cigarette pack-years and current use of other tobacco products, thus reflecting associations of current tobacco use with oral health independent of smoking history and concurrent tobacco product use.

Cigarette smoking was associated with the incidence of several adverse oral health outcomes (ie, gum disease diagnosis, loose teeth, and 1 or more teeth removed), supporting the previously documented causal relationship between smoking and periodontal disease.^4^ Although current cigarette smoking was not associated with incidence of bone loss around teeth, increasing cigarette pack-year history was significantly associated with this oral health outcome (results in eTable 5 in Supplement 1), confirming the importance of both number of cigarettes smoked and duration of smoking to risk of poor oral health, particularly for outcomes that may take a longer time to manifest.^4^ Over the short period of follow-up in this study, current cigar smoking but not cigarette smoking was positively associated with incidence of precancerous oral lesions, a heterogenous group of potentially malignant disorders.^28^ Future studies can explore whether the inconsistent findings across combustible products (ie, cigarettes, cigars, pipes, and hookah) are possibly due to differences in use behaviors between these products.^29,30,31^

In the current study, current ENDS use was positively associated with bleeding after brushing or flossing, similar to a prior pilot study on gum bleeding in e-cigarette users.^14^ However, determining whether this is a direct effect of ENDS use or the observed increase in bleeding is due to quitting cigarette smoking, especially given that most ENDS users are former or current cigarette users,^32^ is an area for future research. Nevertheless, although limited, the preclinical, clinical, and epidemiological evidence suggests potential oral health harms associated with ENDS use compared with never using,^15,33,34^ possibly via dental and periodontal damage, cellular changes, or alteration of the oral microbiome.^33,34^ Although this study did not find associations between current ENDS use and incidence of other oral health outcomes, it focused on short-term changes in oral health. Short-term changes in oral health (eg, gum bleeding) may be easier to detect than long-term changes such as bone loss around teeth or precancerous oral lesions; therefore, future analyses using additional waves of the PATH Study can explore incidence of oral health outcomes that may be associated with longer term ENDS use.

Smokeless tobacco use is associated with precancerous oral lesions in South Asia, where these products are designed to be highly addictive and their use is culturally and socially acceptable and often combined with other carcinogens (eg, areca nut).^35^ A recent review of systematic reviews^36^ concluded there is a positive association between smokeless tobacco use and oral cancer worldwide. However, associations varied by geographic region such that studies in North America showed no associations with oral cancer.^36^ Although snus is a recent addition to the US tobacco market, 8 snus products received modified risk marketing authorization from the FDA.^37,38^ Previous studies^37,39^ have shown considerable heterogeneity in composition, including pH, nicotine, and tobacco-specific nitrosamines, among snus varieties, particularly between US and European products, suggesting that these products may pose varying degrees of risk. The lack of associations between snus use and incidence of oral health outcomes may reflect this variability or may be due to low statistical power but is consistent with findings from Swedish studies.^40,41^

### Limitations

Limitations of this study include the use of self-reported oral health outcomes that may have underestimated clinical oral disease; however, the measures in this study closely align with self-reported measures from the National Health and Nutrition Examination Survey that have demonstrated adequate internal validity.^18^ In addition, incidence of gum disease, precancerous oral lesions, and bone loss may be underestimated as a result of classifying participants who were not asked these questions at W4 and W5 (because they had not seen a dentist in the past 12 months) as not having the outcome. Although this study examined associations of tobacco products with oral health outcomes mutually adjusted for each other, future studies can examine associations between exclusive or polytobacco use and oral health. The study’s focus on current tobacco use limited our ability to control for past tobacco use aside from cigarette pack-years. Because of the limited sample sizes, analyses of specific FDA-authorized, modified-risk snus products and oral health outcomes were not feasible. Additionally, unmeasured oral health confounders (eg, sugary diet and oral hygiene habits such as flossing or brushing, which were available only in later waves) had unknown impacts on our findings. Analyses were restricted to participants who responded to all 5 waves because of the PATH Study weights available; however, nonresponse bias analyses showed that the longitudinal weighting adjustments satisfactorily addressed potential bias due to loss to follow-up.^21^ Furthermore, the shift from annual to biennial data collection between W4 and W5 resulted in a longer period of assessment of oral health outcomes at W5.

## Conclusions

This nationally representative cohort study confirmed associations of current combustible tobacco use with incidence of adverse oral health outcomes and also showed an association between current ENDS use and incidence of bleeding after brushing or flossing. In addition to informing FDA’s tobacco regulatory actions, the findings emphasize the continued importance of tobacco cessation counseling and resources in clinical practice.

## Notes

Supplement 1. **eTable 1.** Construct, Variable Descriptions and Categories **eTable 2.** Univariate Cox Proportional Hazards Models Assessing Associations of Tobacco Product Use and Covariates With Incidence of Gum Disease and Precancerous Oral Lesions, PATH Study **eTable 3.** Univariate Cox Proportional Hazards Models Assessing Associations of Tobacco Product Use and Covariates With Incidence of Bone Loss Around Teeth, Bleeding After Brushing/Flossing, Loose Teeth, and One or More Teeth Removed, PATH Study **eTable 4.** Cox Proportional Hazards Model With Covariates Assessing Associations of Tobacco Product Use With Incidence of Gum Disease and Precancerous Oral Lesions, PATH Study **eTable 5.** Cox Proportional Hazards Model With Covariates Assessing Associations of Tobacco Product Use With Incidence of Bone Loss Around Teeth, Bleeding After Brushing/Flossing, Loose Teeth, and One or More Teeth Removed, PATH Study

Supplement 2.
Data Sharing Statement
