# Supplementary material for: Tobacco Use and Incidence of Adverse Oral Health Outcomes Among US Adults in the Population Assessment of Tobacco and Health Study
Source: JAMA Netw Open. 2022 Dec 9;5(12):e2245909. doi: 10.1001/jamanetworkopen.2022.45909 (PMC9856400; doi:10.1001/jamanetworkopen.2022.45909)
Supplement: Supplement 1. — eTable 1. Construct, Variable Descriptions and Categories eTable 2. Univariate Cox Proportional Hazards Models Assessing Associations of Tobacco Product Use and Covariates With Incidence of Gum Disease and Precancerous Oral Lesions, PATH Study eTable 3. Univariate Cox Proportional Hazards Models Assessing Associations of Tobacco Product Use and Covariates With Incidence of Bone Loss Around Teeth, Bleeding After Brushing/Flossing, Loose Teeth, and One or More Teeth Removed, PATH Study eTable 4. Cox Proportional Hazards Model With Covariates Assessing Associations of Tobacco Product Use With Incidence of Gum Disease and Precancerous Oral Lesions, PATH Study eTable 5. Cox Proportional Hazards Model With Covariates Assessing Associations of Tobacco Product Use With Incidence of Bone Loss Around Teeth, Bleeding After Brushing/Flossing, Loose Teeth, and One or More Teeth Removed, PATH Study [file jamanetwopen-e2245909-s001.pdf]

Supplemental Online Content

Silveira ML, Everard CD, Sharma E, et al. Tobacco use and incidence of adverse oral health outcomes among US adults in the Population Assessment of Tobacco and Health Study. *JAMA Netw Open*. 2022;5(12):e2245909.
doi:10.1001/jamanetworkopen.2022.45909

eTable 1. Construct, Variable Descriptions and Categories

eTable 2. Univariate Cox Proportional Hazards Models Assessing Associations of Tobacco Product Use and Covariates With Incidence of Gum Disease and Precancerous Oral Lesions, PATH Study

eTable 3. Univariate Cox Proportional Hazards Models Assessing Associations of Tobacco Product Use and Covariates With Incidence of Bone Loss Around Teeth, Bleeding After Brushing/Flossing, Loose Teeth, and One or More Teeth Removed, PATH Study

eTable 4. Cox Proportional Hazards Model With Covariates Assessing Associations of Tobacco Product Use With Incidence of Gum Disease and Precancerous Oral Lesions, PATH Study

eTable 5. Cox Proportional Hazards Model With Covariates Assessing Associations of Tobacco Product Use With Incidence of Bone Loss Around Teeth, Bleeding After Brushing/Flossing, Loose Teeth, and One or More Teeth Removed, PATH Study

This supplemental material has been provided by the authors to give readers additional information about their work.

© 2022 Silveira ML et al. *JAMA Network Open.*

eTable 1. Construct, Variable Descriptions and Categories

Construct	PATH Study Variable name	Variable definition	ASK specification	Label[a]
Wave 1 current established cigarette use	R01R_A_CUR_ESTD_CIGS	Has ever smoked a cigarette, has smoked at least 100 cigarettes in lifetime, and currently smokes every day or some days.	All adult respondents	1=Yes<br>2=No
Wave 2 current established cigarette use	R02R_A_CUR_ESTD_CIGS	Wave 2 Adult respondents who have smoked at least 100 cigarettes in their lifetime, and currently smoke every day or some days.	All adult respondents	1=Yes<br>2=No
Wave 3 current established cigarette use	R03R_A_CUR_ESTD_CIGS	Wave 3 Adult respondents who have smoked at least 100 cigarettes in their lifetime, and currently smoke every day or some days.	All adult respondents	1=Yes<br>2=No
Wave 4 current established cigarette use	R04R_A_CUR_ESTD_CIGS	Wave 4 adult respondents who have smoked at least 100 cigarettes in their lifetime, and currently smoke every day or some days	All adult respondents	1=Yes<br>2=No
Wave 1 current established ENDS use	R01R_A_CUR_ESTD_ECIG	Has ever used an e-cigarette, has used fairly regularly, and uses every day or some days.	All adult respondents	1=Yes<br>2=No
Wave 2 current established ENDS use	R02R_A_CUR_ESTD_EPRODS	Wave 2 Adult respondents who have ever used any electronic nicotine products fairly regularly, and currently use every day or some days.	All adult respondents	1=Yes<br>2=No

© 2022 Silveira ML et al. *JAMA Network Open.*

Wave 3 current established ENDS use	R03R_A_CUR_ESTD_EPRODS	Wave 3 Adult respondents who have ever used any electronic nicotine products, have ever used them fairly regularly, and currently use them every day or some days.	All adult respondents	1=Yes<br>2=No
Wave 4 current established ENDS use	R04R_A_CUR_ESTD_EPRODS	Wave 4 adult respondents who have ever used any electronic nicotine products, have ever used them fairly regularly, and currently use every day or some days.	All adult respondents	1=Yes<br>2=No
Wave 1 current established cigar use	R01R_A_CUR_ESTD_CIGAR	Wave 1 Adult respondents who are current established traditional cigar, cigarillo or filtered cigar smokers.<br>Has ever smoked traditional cigars, has smoked fairly regularly, and smokes every day or some days.<br>Has ever smoked cigarillos, has smoked fairly regularly, and smokes every day or some days.<br>Has ever smoked filtered cigars, has smoked fairly regularly, and smokes every day or some days.	All adult respondents	1=Yes<br>2=No
Wave 2 current established cigar use	R02R_A_CUR_ESTD_CIGAR	Wave 2 Adult respondents who have ever smoked any cigar, have smoked fairly regularly, and currently smoke every day or some days.	All adult respondents	1=Yes<br>2=No

© 2022 Silveira ML et al. *JAMA Network Open.*

Wave 3 current established cigar use	R03R_A_CUR_ESTD_CIGAR	Wave 3 Adult respondents who have ever smoked a cigar, have smoked at least one cigar product fairly regularly, and currently smoke every day or some days.	All adult respondents	1=Yes<br>2=No
Wave 4 current established cigar use	R04R_A_CUR_ESTD_CIGAR	Wave 4 adult respondents who have smoked a cigar, have ever smoked at least one cigar product fairly regularly, and currently smoke every day or some days.	All adult respondents	1=Yes<br>2=No
Wave 1 current established pipe use	R01R_A_CUR_ESTD_PIPE	Has ever smoked a pipe, has smoked fairly regularly, and smokes every day or some days.	All adult respondents	1=Yes<br>2=No
Wave 2 current established pipe use	R02R_A_CUR_ESTD_PIPE	Wave 2 Adult respondents who have ever smoked a pipe, have ever smoked fairly regularly, and currently smoke every day or some days.	All adult respondents	1=Yes<br>2=No
Wave 3 current established pipe use	R03R_A_CUR_ESTD_PIPE	Wave 3 Adult respondents who have ever smoked a pipe, have ever smoked fairly regularly, and currently smoke every day or some days.	All adult respondents	1=Yes<br>2=No
Wave 4 current established pipe use	R04R_A_CUR_ESTD_PIPE	Wave 4 adult respondents who have ever smoked a pipe, have ever smoked them fairly regularly, and currently smoke every day or some days.	All adult respondents	1=Yes<br>2=No

© 2022 Silveira ML et al. *JAMA Network Open.*

Wave 1 current established hookah use	R01R_A_CUR_ESTD_HOOK	Has ever smoked a hookah, has smoked fairly regularly, and smokes every day or some days.	All adult respondents	1=Yes<br>2=No
Wave 2 current established hookah use	R02R_A_CUR_ESTD_HOOK	Wave 2 Adult respondents who have ever smoked a hookah, have ever smoked fairly regularly, and currently smoke every day or some days.	All adult respondents	1=Yes<br>2=No
Wave 3 current established hookah use	R03R_A_CUR_ESTD_HOOK	Wave 3 Adult respondents who have ever smoked a hookah, have ever smoked them fairly regularly, and currently smoke every day or some days.	All adult respondents	1=Yes<br>2=No
Wave 4 current established hookah use	R04R_A_CUR_ESTD_HOOK	Wave 4 adult respondents who have ever smoked a hookah, have ever smoked them fairly regularly, and currently smoke every day or some days.	All adult respondents	1=Yes2=No
Wave 1 current established snus use	R01R_A_CUR_ESTD_SNUS	Has ever used snus, has used fairly regularly, and uses every day or some days.	All adult respondents	1=Yes<br>2=No
Wave 2 current established snus use	R02R_A_CUR_ESTD_SNUS	Wave 2 Adult respondents who have ever used snus pouches, have ever used fairly regularly, and currently use every day or some days.	All adult respondents	1=Yes<br>2=No
Wave 3 current established snus use	R03R_A_CUR_ESTD_SNUS	Wave 3 Adult respondents who have ever used snus pouches, have ever used them fairly regularly, and currently use every day or some days.	All adult respondents	1=Yes<br>2=No

© 2022 Silveira ML et al. *JAMA Network Open.*

Wave 4 current established snus use	R04R_A_CUR_ESTD_SNUS	Wave 4 adult respondents who have ever used snus pouches, have ever used them fairly regularly, and currently use every day or some days.	All adult respondents	1=Yes<br>2=No
Wave 1 current established smokeless tobacco use	R01R_A_CUR_ESTD_SMKLS	Has ever used smokeless tobacco, has used fairly regularly, and uses every day or some days.	All adult respondents	1=Yes<br>2=No
Wave 2 current established smokeless tobacco use	R02R_A_CUR_ESTD_SMKLS	Wave 2 Adult respondents who have ever used smokeless tobacco, have ever used fairly regularly, and currently use every day or some days	All adult respondents	1=Yes<br>2=No
Wave 3 current established smokeless tobacco use	R03R_A_CUR_ESTD_SMKLS	Wave 3 Adult respondents who have ever used smokeless tobacco, have ever used it fairly regularly, and currently use every day or some days	All adult respondents	1=Yes<br>2=No
Wave 4 current established smokeless tobacco use	R04R_A_CUR_ESTD_SMKLS	Wave 4 adult respondents who have ever used smokeless tobacco, have ever used it fairly regularly, and currently use every day or some days.	All adult respondents	1=Yes<br>2=No
Wave 1 past 30-day alcohol use	R01_AX0075	In the past 30 days, on how many days did you have one or more alcoholic drinks? Count a drink as a can or bottle of beer; a wine cooler or a glass of wine, champagne, or sherry; a shot of liquor or a mixed drink or cocktail.	Adult respondents who have ever used alcohol and have used alcohol in the past 30 days	Count of number of days (continuous)

© 2022 Silveira ML et al. *JAMA Network Open.*

Wave 2 past 30-day alcohol use	R02_AX0075	In the past 30 days, on how many days did you have one or more alcoholic drinks? A drink is a can or bottle of beer; a wine cooler or a glass of wine, champagne, or sherry; a shot of liquor or a mixed drink or cocktail.	Adult respondents who have used alcohol in the past 12 months and have used alcohol in the past 30 days.	Count of number of days (continuous)
Wave 3 past 30-day alcohol use	R03_AX0075	In the past 30 days, on how many days did you have one or more alcoholic drinks? A drink is a can or bottle of beer; a wine cooler or a glass of wine, champagne, or sherry; a shot of liquor or a mixed drink or cocktail.	Adult respondents who have used alcohol in the past 12 months and have used alcohol in the past 30 days.	Count of number of days (continuous)
Wave 4 past 30-day alcohol use	R04_AX0075	In the past 30 days, on how many days did you have one or more alcoholic drinks? A drink is a can or bottle of beer; a wine cooler or a glass of wine, champagne, or sherry; a shot of liquor or a mixed drink or cocktail.	Continuing and aged-up adult respondents who have used alcohol in the past 12 months and have used alcohol in the past 30 days, or new cohort adult respondents who have ever used alcohol and have used alcohol in the past 12 months and have used alcohol in the past 30 days.	Count of number of days (continuous)
Flossing in the past 7 days at Wave 3	R01_AX0076	On average, on those [days that you drank in the past 30 days / NUMBER OF DAYS FROM AX0075 days], how many alcoholic drinks did you usually have each day? Count a drink as a can or bottle of beer; a wine cooler or a glass of wine,	Respondents who have ever used alcohol (R01_AX0084 = 1 AND (VALID AGE IN R01_AX0074 OR R01_AX0270>0)) and have used alcohol in the past 30 days (R01_AX0073=1), who drank alcohol one or more days (R01_AX0075 != 0).	Count of number of drinks (continuous)

© 2022 Silveira ML et al. *JAMA Network Open.*

		champagne, or sherry; a shot of liquor or a mixed drink or cocktail.		
Wave 2 number of drinks per day	R02_AX0076	On average, on those [days that you drank in the past 30 days / NUMBER OF DAYS FROM R02_AX0075 days], how many alcoholic drinks did you usually have each day? Count a drink as a can or bottle of beer; a wine cooler or a glass of wine, champagne, or sherry; a shot of liquor or a mixed drink or cocktail.	Adult respondents who have used alcohol in the past 12 months and have used alcohol in the past 30 days and had an alcoholic drink on one or more of the past 30 days or did not know or refused to report on how many of the last 30 days they had one or more alcoholic drinks.	Count of number of drinks (continuous)
Wave 3 number of drinks per day	R03_AX0076	On average, on those [days that you drank in the past 30 days / NUMBER OF DAYS FROM R03_AX0075 days], how many alcoholic drinks did you usually have each day? Count a drink as a can or bottle of beer; a wine cooler or a glass of wine, champagne, or sherry; a shot of liquor or a mixed drink or cocktail.	Adult respondents who have used alcohol in the past 12 months and have used alcohol in the past 30 days and had an alcoholic drink on one or more of the past 30 days or did not know or refused to report on how many of the last 30 days they had one or more alcoholic drinks.	Count of number of drinks (continuous)
Wave 4 number of drinks per day	R04_AX0076	On average, on those [days that you drank in the past 30 days / NUMBER OF DAYS FROM R04_AX0075 days], how many alcoholic drinks did you usually have each day? Count a drink as a can or bottle of beer; a wine cooler or a glass of wine, champagne, or sherry; a shot of liquor or a mixed drink or cocktail.	Continuing and aged-up adult respondents who have used alcohol in the past 12 months and have used alcohol in the past 30 days and had an alcoholic drink on one or more of the past 30 days or did not know or refused to report on how many of the last 30 days they had one or more alcoholic	Count of number of drinks (continuous)

© 2022 Silveira ML et al. *JAMA Network Open.*

			drinks, or new cohort adult respondents who have ever used alcohol and have used alcohol in the past 12 months and have used alcohol in the past 30 days and had an alcoholic drink on one or more of the past 30 days or did not know or refused to report on how many of the last 30 days they had one or more alcoholic drinks.	
Wave 1 past 30-day marijuana use	R01R_A_P30D_MJ	Wave 1 Adult who has smoked marijuana within the past 30 days.	All adult respondents	1=Yes<br>2=No
Wave 2 past 30-day marijuana use	R02R_A_MJ_PM	Wave 2 adult respondents who have smoked marijuana within the past month.	All adult respondents	1=Yes<br>2=No
Wave 3 past 30-day marijuana use	R03R_A_MJ_PM	Wave 3 adult respondents who have used marijuana within the past month.<br><br>Note: Values of -99911 were recoded as 2=No as all of these respondents reported “2=no” to R03_AX0675 (In past 30 days, used marijuana, hash, THC, grass, pot or weed) or R03_AX0085_12M (In past 12 months, used marijuana, hash, THC, grass, pot or weed).	All adult respondents	1=Yes<br>2=No

© 2022 Silveira ML et al. *JAMA Network Open.*

Wave 4 past 30-day marijuana use	R04R_A_MJ_PM	Wave 4 adult respondents who have smoked marijuana within the past month.	All adult respondents	1=Yes<br>2=No
Lifetime history of gum disease at Wave 1	R01_AX0129	Have you ever been told by a dentist, hygienist, or other health professional that you have gum disease?	All adult respondents	1=Yes<br>2=No
Wave 2 past 12-month gum disease	R02_AX0129_12M	In the past 12 months, have you been told by a dentist, hygienist, or other health professional that you have gum disease?	Continuing adult respondents	1=Yes<br>2=No
Wave 3 past 12-month gum disease	R03_AX0129_12M	In the past 12 months, have you been told by a dentist, hygienist, or other health professional that you have gum disease?	Continuing adult respondents	1=Yes<br>2=No

© 2022 Silveira ML et al. *JAMA Network Open.*

Wave 4 past 12-month gum disease	R04_AX0129_12M**	In the past 12 months, have you been told by a dentist, hygienist, or other health professional that you have gum disease?<br><br>Note: These items were skipped incorrectly by continuing adult respondents (R04_ADULTTYPE = 1) who did not know or refused to report if they had seen a dentist in the past 12 months (R04_AX0002 = -8, -7). This issue was fixed in a field update on 01/31/2017.	Continuing adult respondents who have seen a dentist in the past 12 months	1=Yes<br>2=No
Wave 5 past 12-month gum disease	R05_AX0129_12M	In the past 12 months, have you been told by a dentist, hygienist, or other health professional that you have gum disease?	Continuing adult respondents who have seen a dentist in the past 12 months or did not know or refused to report whether they have seen a dentist in the past 12 months	1=Yes<br>2=No
Lifetime history of precancerous oral lesions at Wave 1	R01_AX0132	Have you ever been told by a doctor, dentist, or other health professional that you have pre-cancerous oral lesions?	All adult respondents	1=Yes<br>2=No
Wave 2 past 12-month precancerous oral lesions	R02_AX0132_12M	In the past 12 months, have you been told by a doctor, dentist, or other health professional that you have pre-cancerous oral lesions	Continuing adult respondents	1=Yes2=No

© 2022 Silveira ML et al. *JAMA Network Open.*

Wave 3 past 12-month precancerous oral lesions	R03_AX0132_12M	In the past 12 months, have you been told by a doctor, dentist, or other health professional that you have pre-cancerous oral lesions?	Continuing adult respondents	1=Yes<br>2=No
Wave 4 past 12-month precancerous oral lesions	R04_AX0132_12M**	In the past 12 months, have you been told by a doctor, dentist, or other health professional that you have pre-cancerous oral lesions?<br><br>Note: These items were skipped incorrectly by continuing adult respondents (R04_ADULTTYPE = 1) who did not know or refused to report if they had seen a dentist in the past 12 months (R04_AX0002 = -8, -7). This issue was fixed in a field update on 01/31/2017.	Continuing adult respondents who have seen a dentist in the past 12 months	1=Yes<br>2=No
Wave 5 past 12-month precancerous oral lesions	R05_AX0132_12M	In the past 12 months, have you been told by a doctor, dentist, or other health professional that you have pre-cancerous oral lesions?	Continuing adult respondents who have seen a dentist in the past 12 months or did not know or refused to report whether they have seen a dentist in the past 12 months	1=Yes<br>2=No
Lifetime history of bone loss at Wave 3	R03_AX0727	Have you ever been told by a dentist, hygienist, or other health professional that you lost bone around your teeth?	All adult respondents	1=Yes<br>2=No

© 2022 Silveira ML et al. *JAMA Network Open.*

Wave 4 past 12-month bone loss	R04_AX0727_12M**	In the past 12 months, have you been told by a dentist, hygienist, or other health professional that you lost bone around your teeth?<br><br>Note: These items were skipped incorrectly by continuing adult respondents (R04_ADULTTYPE = 1) who did not know or refused to report if they had seen a dentist in the past 12 months (R04_AX0002 = -8, -7). This issue was fixed in a field update on 01/31/2017.	Continuing adult respondents who have seen a dentist in the past 12 months	1=Yes<br>2=No
Wave 5 past 12-month bone loss	R05_AX0727_12M	In the past 12 months, have you been told by a dentist, hygienist, or other health professional that you lost bone around your teeth?	Continuing adult respondents who have seen a dentist in the past 12 months or did not know or refused to report whether they have seen a dentist in the past 12 months	1=Yes<br>2=No
Lifetime history of bleeding after brushing/flossing at Wave 3	R03_AX0693	Have you ever observed any bleeding after brushing or flossing, or due to other conditions in your mouth?	All adult respondents	1=Yes<br>2=No
Wave 4 past 12-month bleeding after brushing/flossing	R04_AX0693_12M	In the past 12 months, have you observed any bleeding after brushing or flossing, or due to other conditions in your mouth?	Continuing adult respondents	1=Yes<br>2=No
Wave 5 past 12-month bleeding after brushing/flossing	R05_AX0693_12M	In the past 12 months, have you observed any bleeding after brushing or flossing, or due to other conditions in your mouth?	Continuing adult respondents	1=Yes<br>2=No

© 2022 Silveira ML et al. *JAMA Network Open.*

Lifetime history of loose teeth at Wave 3	R03_AX0694	Have you ever had any teeth become loose on their own, without an injury?	All adult respondents	1=Yes<br>2=No
Wave 4 past 12-month loose teeth	R04_AX0694_12M	In the past 12 months, have you had any teeth become loose on their own, without an injury?	Continuing adult respondents	1=Yes<br>2=No
Wave 5 past 12-month loose teeth	R05_AX0694_12M	In the past 12 months, have you had any teeth become loose on their own, without an injury?	Continuing adult respondents whose permanent teeth have not all been removed because of tooth decay or gum disease or did not know or refused to report how many of their permanent teeth have been removed because of tooth decay or gum disease	1=Yes2=No
Lifetime history of number of teeth removed at Wave 3	R03_AX0695	How many of your permanent teeth have been removed because of tooth decay or gum disease? Do not include teeth lost for other reasons, such as injury or orthodontics.	All adult respondents	0=0<br>1= 1 or more
Wave 4 past 12-month number of teeth removed	R04_AX0695_12M	In the past 12 months, how many of your permanent teeth have been removed because of tooth decay or gum disease? Do not include teeth lost for other reasons, such as injury or orthodontics.	Continuing adult respondents	0=0<br>1=1 or more

© 2022 Silveira ML et al. *JAMA Network Open.*

Wave 5 past 12-month number of teeth removed	R05_AX0695_12M	In the past 12 months, how many of your permanent teeth have been removed because of tooth decay or gum disease? Include teeth lost to infection, but do not include teeth lost for other reasons, such as injury or orthodontics.	Continuing adult respondents	1= None<br>2= 1 to 5<br>3= 6 or more, but not all<br>4= All<br><br>NOTE: This variable was recoded as 0=0 1=1 or more
Age at Wave 1	R01R_A_AGECAT6	Wave 1 Adult respondents who are between the ages of 18 and 24, 25 and 34, 35 and 44, 45 and 54, 55 and 64, or 65 and older.	All adult respondents	1= 18 and 24<br>2= 25 and 34<br>3= 35 and 44<br>4= 45 and 54<br>5= 55 and 64<br>6= 65 and older
Age at Wave 3	R03R_A_AGECAT6	Wave 3 adult respondents who are between the ages of 18 and 24, 25 and 34, 35 and 44, 45 and 54, 55 and 64, or 65 and older	All adult respondents	1= 18 and 24<br>2= 25 and 34<br>3= 35 and 44<br>4= 45 and 54<br>5= 55 and 64<br>6= 65 and older
Sex at Wave 1	R01R_A_SEX_IMP	Imputed Sex	All adult respondents	1= Male<br>2=Female
Sex at Wave 3	R03R_A_SEX	Wave 3 Adult sex from the interview.	All adult respondents	1= Male<br>2=Female
Race/ethnicity at Wave 1	R01R_A_ETHRACEC AT4_IMP	Wave 1 adult reported race/ethnicity: non-Hispanic white, non-Hispanic black, non-Hispanic other race including multi-racial, or Hispanic	All adult respondents	1 = Non-Hispanic, White<br>2 = Non-Hispanic, Black<br>3 = Non-Hispanic other race including multi-racial<br>4 = Hispanic

© 2022 Silveira ML et al. *JAMA Network Open.*

Race/ethnicity at Wave 3	R03R_A_ETHRACECAT 4	Wave 3 adult reported race/ethnicity: non-Hispanic white, non-Hispanic black, non-Hispanic other race including multi-racial, or Hispanic.	All adult respondents	1 = Non-Hispanic, White 2 = Non-Hispanic, Black 3 = Non-Hispanic other race including multi-racial 4 = Hispanic
Education at Wave 1	R01R_A_EDUC_CAT5	Wave 1 Adults with less than high school or some high school (no diploma) education, a GED, a high school diploma, some college or associate degree, or a bachelor's degree or higher.	All adult respondents	1 = Less than high school or some high school, no diploma<br>2 = GED<br>3 = High school graduate - diploma<br>4 = Some college (no degree) or associate degree<br>5 = Bachelor's degree or more
Education at Wave 3	R03R_A_EDUC_CAT 5	Wave 3 Adults with less than high school or some high school (no diploma) education, a GED, a high school diploma, some college or associate degree, or a bachelor's degree or higher.	All adult respondents	1 = Less than high school or some high school, no diploma<br>2 = GED<br>3 = High school graduate - diploma<br>4 = Some college (no degree) or associate degree<br>5 = Bachelor's degree or more
Household income at Wave 1	R01R_A_INCOMECAT4	Wave 1 adult respondents whose total household income was less than $25,000, $25,000-$49,000, $50,000$74,999, or $75,000 or more within the past 12 months.	All adult respondents	1= Less than $25,000<br>2= $25,000-$49,000<br>3= $50,000-$74,999<br>4= $75,000 or more

© 2022 Silveira ML et al. *JAMA Network Open.*

Household income at Wave 3	R03R_A_INCOMECAT4	Wave 3 adult respondents whose total household income was less than $25,000, $25,000-$49,000, $50,000$74,999, or $75,000 or more within the past 12 months.	All adult respondents	1= Less than $25,000<br>2= $25,000-$49,000<br>3= $50,000-$74,999<br>4= $75,000 or more
Cigarette pack-years at Wave 1	R01R_A_PACKYEARS_CIGS	Wave 1 adult number of cigarette packs smoked per day multiplied by the number of years they have smoked fairly regularly. If respondent has not smoked fairly regularly but reported smoking at least 1 puff, approximate pack year values were assigned.	Adult ever cigarette smokers	Continuous<br><br>Note: This variable was categorized as described in Table 1.
Cigarette pack-years at Wave 3	R03R_A_PACKYEARS_CIGS	Wave 3 adult number of cigarette packs smoked per day multiplied by the number of years they have smoked fairly regularly.	Adult ever cigarette smokers	Continuous<br><br>Note: This variable was categorized as described in Table 1.
Lifetime history of diabetes at Wave 1	R01_AX0281	Have you ever been told by a doctor or other health professional that you have diabetes, sugar diabetes, high blood sugar, or borderline diabetes?	All adult respondents	1=Yes2=No
Lifetime history of diabetes at Wave 3	R03R_A_EVR_DIABETES	Wave 3 adult respondents who have ever told by a doctor, nurse or other health professional that they had diabetes, sugar diabetes, high blood sugar, or borderline diabetes	Adult respondents who have ever told by a doctor, nurse or other health professional that they had diabetes, sugar diabetes, high blood sugar, or borderline diabetes	1=Yes<br>2=No

© 2022 Silveira ML et al. *JAMA Network Open.*

Flossing in the past 7 days at Wave 3	R03_AX0696	Aside from brushing your teeth with a toothbrush, in the last seven days, how many times did you use dental floss or any other device to clean between your teeth?	All adult respondents	1= Yes<br>2= No
[a]Variables were categorized as described in Table 1.				

© 2022 Silveira ML et al. *JAMA Network Open.*

eTable 2: Univariate Cox Proportional Hazards Models Assessing Associations of Tobacco Product Use and Covariates With Incidence of Gum Disease and Precancerous Oral Lesions, PATH Study

	Incidence of oral health outcomes from Waves 2 to 5						
	Gum disease[a]				Precancerous oral lesions[b]		
Covariate[c]	HR[d]	95% CI[d]	p-value		HR[d]	95% CI[d]	p-value
Time-dependent variables from Waves 1 to 4							
Current established cigarette use[e]	**1.53**	**(1.30, 1.79)**	**<.0001**		**2.06**	**(1.40, 3.03)**	**<.001**
Current established ENDS use[e]	**1.43**	**(1.12, 1.83)**	**<.01**		0.83	(0.39, 1.77)	0.63
Current established cigar use[e]	1.33	(1.00, 1.78)	0.05		**3.14**	**(2.00, 4.93)**	**<.0001**
Current established pipe use[e]	1.02	(0.47, 2.20)	0.96		1.65	(0.48, 5.67)	0.42
Current established hookah use[e]	**1.78**	**(1.22, 2.60)**	**<.01**		2.72	(1.02, 7.23)	0.05
Current established smokeless tobacco use[e]	0.90	(0.65, 1.24)	0.51		**2.45**	**(1.38, 4.34)**	**<.01**
Current established snus use[e]	0.91	(0.28, 2.92)	0.87		1.68	(0.36, 7.90)	0.51
Heavy alcohol use[e]	1.01	(0.76, 1.36)	0.93		0.52	(0.26, 1.03)	0.06
Past 30-day marijuana use[e]	1.20	(0.95, 1.51)	0.13		0.97	(0.62, 1.54)	0.91
Baseline covariates at Wave 1							
Sex							
Male	Ref.				Ref.		
Female	1.10	(0.94, 1.29)	0.22		0.64	(0.42, 0.99)	0.05
Age							
18-24	Ref.				Ref.		
25-34	**1.51**	**(1.21, 1.88)**	**<.001**		1.16	(0.63, 2.11)	0.63
35-44	1.18	(0.91, 1.52)	0.21		0.90	(0.48, 1.71)	0.75
45-54	**1.49**	**(1.17, 1.90)**	**<.01**		1.43	(0.76, 2.68)	0.26
55-64	1.30	(0.97, 1.73)	0.08		0.81	(0.42, 1.55)	0.51
65+	1.24	(0.83, 1.85)	0.29		0.61	(0.23, 1.62)	0.32
Race/Ethnicity							
Non-Hispanic, White	Ref.				Ref.		
Non-Hispanic, Black	1.20	(0.92, 1.56)	0.17		**1.79**	**(1.07, 3.00)**	**0.03**

© 2022 Silveira ML et al. *JAMA Network Open.*

Non-Hispanic other, including multi-racial	1.07	(0.69, 1.67)	0.76		0.71	(0.26, 1.92)	0.50
Hispanic	1.11	(0.88, 1.38)	0.37		1.38	(0.84, 2.25)	0.20
Educational attainment							
Less than high school or some high school, no diploma	Ref.				Ref.		
GED	1.01	(0.67, 1.52)	0.98		**0.47**	**(0.24, 0.92)**	**0.03**
High school graduate - diploma	1.01	(0.72, 1.41)	0.95		**0.54**	**(0.32, 0.92)**	**0.02**
Some college (no degree) or associate degree	0.94	(0.71, 1.25)	0.65		**0.28**	**(0.16, 0.48)**	**<.0001**
Bachelor's degree or more	0.83	(0.61, 1.13)	0.23		**0.11**	**(0.05, 0.25)**	**<.0001**
Annual household income							
Less than $25,000	Ref.				Ref.		
$25,000-$49,999	0.93	(0.75, 1.15)	0.49		**0.38**	**(0.21, 0.70)**	**<.01**
$50,000-$74,999	0.85	(0.66, 1.10)	0.21		0.53	(0.23, 1.23)	0.14
$75,000 or more	**0.80**	**(0.65, 0.98)**	**0.03**		**0.30**	**(0.16, 0.57)**	**<.001**
Cigarette pack-years							
0	Ref.				Ref.		
>0 to ≤5	**1.30**	**(1.07, 1.60)**	**0.01**		1.21	(0.73, 2.01)	0.46
>5 to ≤10	**1.46**	**(1.11, 1.92)**	**0.01**		0.88	(0.44, 1.78)	0.73
>10 to ≤15	1.31	(0.94, 1.81)	0.11		1.18	(0.45, 3.14)	0.74
>15 to ≤20	**1.74**	**(1.20, 2.52)**	**<.01**		1.09	(0.48, 2.50)	0.83
>20	1.26	(0.99, 1.61)	0.06		1.58	(0.87, 2.88)	0.13
Lifetime history of diabetes[f]	**1.37**	**(1.02, 1.82)**	**0.04**		0.99	(0.52, 1.90)	0.98

[a]Gum disease was defined based on a positive response to the question whether participants were told by a dentist, hygienist, or other health professional that they had gum disease in the past 12 months regardless of missing data at any of the waves. Participants who responded no to this question at Waves 2 and 3 and who either responded no to this question or were not asked the question because of not having seen a dentist in the past 12 months at Waves 4 and 5 are categorized as not having the outcome.

[b]Pre-cancerous oral lesions were defined based on a positive response to the question whether participants were told by a dentist, hygienist, or other health professional that they had pre-cancerous oral lesions in the past 12 months regardless of missing data at any of the waves. Participants who responded no to this question at Waves 2 and 3 and who either responded no to this question or were not asked the question because of not having seen a dentist in the past 12 months at Waves 4 and 5 are categorized as not having the outcome.

[c]See supplemental table 1 for variable description.

[d]Unadjusted hazard ratio (HR)s and 95% confidence intervals (CI)s are from univariate survival analysis models weighted using the Wave 5 all-waves weights for the Wave 1 Cohort.

[e]Current established use (yes/no) for cigarettes was defined as having smoked at least 100 cigarettes in one's lifetime and now smokes every day or some days. For other products, current established use (yes/no) was defined as ever using the product "fairly regularly" and now smokes/uses every day or some days. Ref: yes vs. no.

[f]Ref: yes vs. no.

© 2022 Silveira ML et al. *JAMA Network Open.*

eTable 3: Univariate Cox Proportional Hazards Models Assessing Associations of Tobacco Product Use and Covariates With Incidence of Bone Loss Around Teeth, Bleeding After Brushing/Flossing, Loose Teeth, and One or More Teeth Removed, PATH Study

	Incidence of oral health outcomes from Waves 4 to 5											
	Bone loss[a]			Bleeding after brushing/flossing[b]			Loose teeth[c]			One or more teeth removed[d]		
Covariate[e]	HR[f]	95% CI[f]	p-value	HR[f]	95% CI[f]	p-value	HR[f]	95% CI[f]	p-value	HR[f]	95% CI[f]	p-value
Time-dependent variables from Waves 3 to 4												
Current established cigarette use[g]	**1.31**	**(1.09, 1.59)**	**<.01**	**1.17**	**(1.06, 1.29)**	**<.01**	**2.53**	**(2.11, 3.02)**	**<.0001**	**2.23**	**(1.94, 2.55)**	**<.0001**
Current established ENDS use[g]	1.07	(0.77, 1.49)	0.69	**1.75**	**(1.47, 2.09)**	**<.0001**	**1.48**	**(1.12, 1.97)**	**0.01**	1.17	(0.92, 1.49)	0.19
Current established cigar use[g]	1.01	(0.70, 1.48)	0.95	**1.28**	**(1.01, 1.61)**	**0.04**	**2.03**	**(1.47, 2.80)**	**<.0001**	**1.74**	**(1.31, 2.31)**	**<.001**
Current established pipe use[g]	1.46	(0.62, 3.42)	0.38	0.95	(0.39, 2.30)	0.91	2.22	(0.76, 6.51)	0.14	**2.47**	**(1.26, 4.84)**	**0.01**
Current established hookah use[g]	0.89	(0.44, 1.81)	0.74	**1.99**	**(1.48, 2.67)**	**<.0001**	0.88	(0.45, 1.72)	0.70	0.92	(0.53, 1.60)	0.78
Current established smokeless tobacco use[g]	0.67	(0.34, 1.35)	0.26	1.17	(0.91, 1.51)	0.21	1.08	(0.73, 1.61)	0.70	1.31	(0.94, 1.82)	0.11
Current established snus use[g]	0.70	(0.21, 2.32)	0.56	1.04	(0.39, 2.74)	0.94	1.82	(0.82, 4.00)	0.14	1.61	(0.91, 2.84)	0.10
Heavy alcohol use[g]	1.27	(0.91, 1.78)	0.16	**1.29**	**(1.08, 1.55)**	**0.01**	1.25	(0.92, 1.69)	0.16	**0.73**	**(0.54, 0.97)**	**0.03**
Past 30-day marijuana use[g]	1.21	(0.93, 1.59)	0.16	**1.75**	**(1.53, 2.00)**	**<.0001**	**1.60**	**(1.32, 1.93)**	**<.0001**	1.02	(0.85, 1.23)	0.85
Flossed in the past 7 days[h]	**1.37**	**(1.06, 1.77)**	**0.02**	0.98	(0.86, 1.13)	0.80	**0.68**	**(0.57, 0.81)**	**<.0001**	**0.64**	**(0.54, 0.76)**	**<.0001**
Baseline covariates at Wave 3												
Sex												
Male	Ref.i			Ref.[j]			Ref.			Ref.		
Female	1.09	(0.88, 1.35)	0.42	1.05	(0.92, 1.20)	0.49	1.00	(0.83, 1.21)	>.99	0.87	(0.75, 1.01)	0.07
Age												

© 2022 Silveira ML et al. *JAMA Network Open.*

18-24	Ref.			Ref.			Ref.[j]			Ref.		
25-34	**1.89**	**(1.37, 2.60)**	**<.001**	**0.73**	**(0.63, 0.84)**	**<.0001**	0.92	(0.72, 1.17)	0.47	1.17	(0.98, 1.39)	0.08
35-44	**1.97**	**(1.41, 2.76)**	**<.001**	**0.49**	**(0.42, 0.58)**	**<.0001**	0.96	(0.73, 1.26)	0.75	1.07	(0.81, 1.40)	0.64
45-54	**2.74**	**(1.94, 3.85)**	**<.0001**	**0.56**	**(0.48, 0.66)**	**<.0001**	**1.51**	**(1.16, 1.95)**	**<.01**	**1.56**	**(1.27, 1.91)**	**<.0001**
55-64	**2.35**	**(1.68, 3.27)**	**<.0001**	**0.27**	**(0.21, 0.33)**	**<.0001**	**1.56**	**(1.19, 2.04)**	**<.01**	**1.74**	**(1.36, 2.22)**	**<.0001**
65+	**2.65**	**(1.75, 4.02)**	**<.0001**	**0.17**	**(0.13, 0.23)**	**<.0001**	**1.48**	**(1.07, 2.06)**	**0.02**	**2.02**	**(1.48, 2.75)**	**<.0001**
Race/Ethnicity												
Non-Hispanic, White	Ref.			Ref.			Ref.			Ref.		
Non-Hispanic, Black	**1.51**	**(1.16, 1.97)**	**<.01**	1.11	(0.91, 1.35)	0.32	**1.72**	**(1.31, 2.26)**	**<.001**	**2.52**	**(2.06, 3.07)**	**<.0001**
Non-Hispanic other, including multi-racial	1.41	(0.95, 2.10)	0.09	1.30	(1.00, 1.69)	0.05	0.91	(0.60, 1.37)	0.64	1.21	(0.88, 1.65)	0.24
Hispanic	1.08	(0.79, 1.48)	0.62	**1.31**	**(1.08, 1.59)**	**0.01**	1.35	(1.02, 1.79)	0.04	**1.83**	**(1.46, 2.29)**	**<.0001**
Educational attainment												
Less than high school or some high school, no diploma	Ref.			Ref.			Ref.			Ref.		
GED	1.23	(0.71, 2.11)	0.46	0.93	(0.68, 1.26)	0.63	0.94	(0.65, 1.36)	0.74	0.91	(0.65, 1.28)	0.58
High school graduate - diploma	1.20	(0.83, 1.73)	0.33	0.99	(0.77, 1.28)	0.96	**0.60**	**(0.43, 0.86)**	**0.01**	**0.53**	**(0.40, 0.70)**	**<.0001**
Some college (no degree) or associate degree	1.21	(0.85, 1.74)	0.29	1.14	(0.94, 1.39)	0.18	**0.42**	**(0.30, 0.57)**	**<.0001**	**0.31**	**(0.24, 0.39)**	**<.0001**
Bachelor's degree or more	0.95	(0.65, 1.39)	0.80	1.13	(0.90, 1.43)	0.28	**0.20**	**(0.14, 0.29)**	**<.0001**	**0.19**	**(0.14, 0.25)**	**<.0001**
Annual household income												
Less than $25,000	Ref.[i]			Ref.			Ref.			Ref.		
$25,000-$49,999	0.84	(0.65, 1.09)	0.19	**0.82**	**(0.70, 0.95)**	**0.01**	**0.58**	**(0.45, 0.75)**	**<.0001**	**0.56**	**(0.45, 0.68)**	**<.0001**
$50,000-$74,999	1.00	(0.74, 1.36)	0.99	0.84	(0.70, 1.02)	0.08	**0.39**	**(0.27, 0.55)**	**<.0001**	**0.53**	**(0.43, 0.65)**	**<.0001**

© 2022 Silveira ML et al. *JAMA Network Open.*

$75,000 or more	0.78	(0.57, 1.06)	0.11	0.85	(0.72, 1.00)	0.05	**0.20**	**(0.14, 0.29)**	**<.0001**	**0.25**	**(0.20, 0.31)**	**<.0001**
Cigarette pack-years												
0	Ref.			Ref.			Ref.			Ref.		
>0 to ≤5	**1.46**	**(1.11, 1.93)**	**0.01**	**1.27**	**(1.08, 1.50)**	**<.01**	**1.52**	**(1.15, 2.02)**	**<.01**	**1.27**	**(1.03, 1.58)**	**0.03**
>5 to ≤10	1.48	(1.01, 2.18)	0.05	1.16	(0.90, 1.49)	0.26	**2.41**	**(1.74, 3.32)**	**<.0001**	**1.44**	**(1.13, 1.84)**	**<.01**
>10 to ≤15	**2.28**	**(1.47, 3.52)**	**<.001**	1.02	(0.73, 1.42)	0.92	**2.26**	**(1.44, 3.56)**	**<.001**	**1.99**	**(1.38, 2.86)**	**<.001**
>15 to ≤20	**1.79**	**(1.14, 2.81)**	**0.01**	1.17	(0.86, 1.61)	0.31	**1.84**	**(1.18, 2.86)**	**0.01**	**2.12**	**(1.41, 3.18)**	**<.001**
>20	**1.94**	**(1.41, 2.66)**	**<.0001**	**0.56**	**(0.44, 0.70)**	**<.0001**	**2.95**	**(2.14, 4.07)**	**<.0001**	**2.47**	**(1.93, 3.16)**	**<.0001**
Lifetime history of diabetes[h]	**1.44**	**(1.10, 1.87)**	**0.01**	**0.74**	**(0.61, 0.91)**	**<.01**	**1.77**	**(1.34, 2.32)**	**<.0001**	**1.77**	**(1.41, 2.22)**	**<.0001**

[a]Bone loss around teeth was defined based on a positive response to the question whether participants were told by a dentist, hygienist, or other health professional that they had lost bone around their teeth in the past 12 months regardless of missing data at any of the waves. Participants who either responded no to this question or were not asked this question because of not having seen a dentist in the past 12 months at Wave 4 and who either responded no this question or were not asked this question because of not having seen a dentist in the past 12 months or did not know or refused to report whether they have seen a dentist in the past 12 months at Wave 5 were categorized as not having the outcome.

[b]Bleeding after brushing/flossing was defined based on a positive response to the question whether participants had observed any bleeding after brushing or flossing, or due to other conditions in their mouth in the past 12 months regardless of missing data at any of the waves. Participants who responded no to this question at Waves 4 and 5 were categorized as not having the outcome.

[c]Loose teeth was defined based on a positive response to the question whether participants had any teeth become loose on their own, without an injury in the past 12 months regardless of missing data at any of the waves. Participants who responded no to this question at Wave 4 and who either responded no to this question or were not asked this question because of having had all their permanent teeth removed at Wave 5 were categorized as not having the outcome.

[d]One or more teeth removed was defined based on greater than zero teeth removed to the question regarding how many of participants' permanent teeth had been removed because of tooth decay or gum disease in the past 12 months regardless of missing data at any of the waves. Those who responded zero to this question at Waves 4 and 5 were categorized as not having the outcome.

[e]See supplemental table 1 for variable description.

[f]Unadjusted hazard ratio (HR)s and 95% confidence intervals (CI)s are from univariate survival analysis models weighted using the Wave 5 all-waves weights for the Wave 1 Cohort.

[g]Current established use (yes/no) for cigarettes was defined as having smoked at least 100 cigarettes in one's lifetime and now smokes every day or some days. For other products, current established use (yes/no) was defined as ever using the product "fairly regularly" and now smokes/uses every day or some days. Ref: yes vs. no.

[h]Ref: yes vs. no.

[i]Two replicate samples could not be used in the model due to non-convergence.

[j]One replicate sample could not be used in the model due to non-convergence.

© 2022 Silveira ML et al. *JAMA Network Open.*

eTable 4: Cox Proportional Hazards Model With Covariates Assessing Associations of Tobacco Product Use With Incidence of Gum Disease and Precancerous Oral Lesions, PATH Study

	Incidence of oral health outcomes from Waves 2 to 5						
	Gum disease[a]				Precancerous oral lesions[b]		
Covariate[c]	AHR[d]	95% CI[d]	p-value		AHR[d]	95% CI[d]	p-value
Time-dependent variables from Waves 1 to 4							
Current established cigarette use[e]	**1.33**	**(1.11, 1.60)**	**<.01**		1.47	(0.87, 2.48)	0.15
Current established ENDS use[e]	1.15	(0.89, 1.47)	0.28		0.56	(0.26, 1.20)	0.14
Current established cigar use[e]	1.14	(0.83, 1.57)	0.42		**2.18**	**(1.38, 3.43)**	**<.01**
Current established pipe use[e]	0.85	(0.38, 1.86)	0.67		0.77	(0.22, 2.67)	0.68
Current established hookah use[e]	**1.78**	**(1.20, 2.63)**	**<.01**		2.70	(0.91, 7.99)	0.07
Current established smokeless tobacco use[e]	0.88	(0.61, 1.28)	0.50		1.66	(0.90, 3.07)	0.10
Current established snus use[e]	0.90	(0.26, 3.10)	0.87		0.73	(0.16, 3.41)	0.68
Heavy alcohol use[f]	0.93	(0.68, 1.27)	0.65		0.48	(0.23, 1.00)	0.05
Past 30-day marijuana use[f]	1.04	(0.83, 1.31)	0.74		0.66	(0.41, 1.05)	0.08
Baseline covariates at Wave 1							
Sex							
Male	Ref.				Ref.		
Female	1.13	(0.96, 1.34)	0.13		0.68	(0.44, 1.07)	0.09
Age							
18-24	Ref.				Ref.		
25-34	**1.54**	**(1.22, 1.95)**	**<.001**		1.55	(0.83, 2.91)	0.17
35-44	1.25	(0.95, 1.64)	0.12		1.27	(0.67, 2.44)	0.46
45-54	**1.57**	**(1.19, 2.07)**	**<.01**		1.76	(0.88, 3.53)	0.11
55-64	1.36	(0.99, 1.88)	0.06		1.03	(0.51, 2.11)	0.93
65+	1.32	(0.88, 1.99)	0.18		0.70	(0.26, 1.92)	0.48
Race/Ethnicity							
Non-Hispanic, White	Ref.				Ref.		
Non-Hispanic, Black	1.13	(0.85, 1.51)	0.38		1.19	(0.66, 2.16)	0.55
Non-Hispanic other, including multi-racial	1.13	(0.72, 1.78)	0.58		0.77	(0.28, 2.16)	0.62
Hispanic	1.11	(0.86, 1.43)	0.43		0.75	(0.39, 1.46)	0.39

© 2022 Silveira ML et al. *JAMA Network Open.*

Educational attainment							
Less than high school or some high school, no diploma	Ref.				Ref.		
GED	0.98	(0.64, 1.51)	0.93		**0.43**	**(0.22, 0.87)**	**0.02**
High school graduate - diploma	1.10	(0.78, 1.56)	0.58		0.60	(0.33, 1.08)	0.09
Some college (no degree) or associate degree	1.04	(0.76, 1.42)	0.82		**0.32**	**(0.18, 0.57)**	**<.001**
Bachelor's degree or more	0.99	(0.67, 1.46)	0.96		**0.15**	**(0.05, 0.41)**	**<.001**
Annual household income							
Less than $25,000	Ref.				Ref.		
$25,000-$49,999	0.95	(0.77, 1.18)	0.64		**0.49**	**(0.26, 0.90)**	**0.02**
$50,000-$74,999	0.92	(0.69, 1.21)	0.54		0.84	(0.35, 2.03)	0.69
$75,000 or more	0.90	(0.68, 1.18)	0.44		0.61	(0.30, 1.21)	0.16
Cigarette pack-years							
0	Ref.				Ref.		
>0 to ≤5	1.24	(1.00, 1.54)	0.05		1.04	(0.55, 1.94)	0.91
>5 to ≤10	1.21	(0.89, 1.64)	0.21		0.58	(0.25, 1.32)	0.19
>10 to ≤15	1.08	(0.77, 1.52)	0.64		0.77	(0.26, 2.26)	0.63
>15 to ≤20	1.44	(0.96, 2.17)	0.08		0.68	(0.24, 1.91)	0.46
>20	1.07	(0.80, 1.42)	0.67		1.05	(0.40, 2.73)	0.92
Lifetime history of diabetes[f]	1.33	(0.98, 1.80)	0.07		0.94	(0.48, 1.82)	0.84

[a]Gum disease was defined based on a positive response to the question whether participants were told by a dentist, hygienist, or other health professional that they had gum disease in the past 12 months regardless of missing data at any of the waves. Participants who responded no to this question at Waves 2 and 3 and who either responded no to this question or were not asked the question because of not having seen a dentist in the past 12 months at Waves 4 and 5 are categorized as not having the outcome.

[b]Pre-cancerous oral lesions were defined based on a positive response to the question whether participants were told by a dentist, hygienist, or other health professional that they had pre-cancerous oral lesions in the past 12 months regardless of missing data at any of the waves. Participants who responded no to this question at Waves 2 and 3 and who either responded no to this question or were not asked the question because of not having seen a dentist in the past 12 months at Waves 4 and 5 are categorized as not having the outcome.

[c]See supplemental table 1 for variable description.

[d]AHR: adjusted hazard ratio; 95% CI: 95% confidence intervals. Models are weighted using the Wave 5 all-waves weights for the Wave 1 Cohort.

[e]Current established use (yes/no) for cigarettes was defined as having smoked at least 100 cigarettes in one's lifetime and now smokes every day or some days. For other products, current established use (yes/no) was defined as ever using the product "fairly regularly" and now smokes/uses every day or some days. Ref: yes vs. no.

[f]Ref: yes vs. no.

© 2022 Silveira ML et al. *JAMA Network Open.*

eTable 5: Cox Proportional Hazards Model With Covariates Assessing Associations of Tobacco Product Use With Incidence of Bone Loss Around Teeth, Bleeding After Brushing/Flossing, Loose Teeth, and One or More Teeth Removed, PATH Study

	Incidence of oral health outcomes from Waves 4 to 5											
	Bone loss around teeth[a]			Bleeding after brushing/flossing[b]			Loose teeth[c]			One or more teeth removed[d]		
Covariate[e]	AHR[f]	95% CI[f]	p-value	AHR[f]	95% CI[f]	p-value	AHR[f]	95% CI[f]	p-value	AHR[f]	95% CI[f]	p-value
Time-dependent variables from Waves 3 to 4												
Current established cigarette use[g]	0.99	(0.77, 1.27)	0.91	0.94	(0.81, 1.10)	0.43	**1.35**	**(1.05, 1.75)**	**0.02**	**1.43**	**(1.18, 1.74)**	**<.001**
Current established ENDS use[g]	0.95	(0.69, 1.31)	0.75	**1.27**	**(1.04, 1.54)**	**0.02**	1.01	(0.75, 1.35)	0.97	1.03	(0.80, 1.33)	0.81
Current established cigar use[g]	0.85	(0.57, 1.25)	0.40	1.04	(0.82, 1.30)	0.76	1.41	(0.99, 1.99)	0.06	1.26	(0.96, 1.64)	0.09
Current established pipe use[g]	1.41	(0.57, 3.47)	0.46	0.87	(0.35, 2.20)	0.77	1.36	(0.46, 4.05)	0.58	1.69	(0.84, 3.38)	0.14
Current established hookah use[g]	1.04	(0.51, 2.12)	0.92	0.98	(0.70, 1.36)	0.88	0.69	(0.36, 1.33)	0.26	0.85	(0.48, 1.50)	0.56
Current established smokeless tobacco use[g]	0.72	(0.33, 1.57)	0.41	1.02	(0.81, 1.28)	0.89	0.86	(0.54, 1.38)	0.53	1.13	(0.79, 1.63)	0.49
Current established snus use[g]	0.95	(0.23, 4.00)	0.94	0.77	(0.32, 1.85)	0.56	1.55	(0.68, 3.56)	0.29	1.26	(0.69, 2.31)	0.45
Heavy alcohol use[h]	1.20	(0.85, 1.68)	0.29	1.17	(0.97, 1.42)	0.10	1.18	(0.84, 1.66)	0.33	0.74	(0.55, 1.00)	0.05
Past 30-day marijuana use[h]	1.25	(0.94, 1.67)	0.13	**1.20**	**(1.05, 1.38)**	**0.01**	1.24	(0.99, 1.54)	0.06	0.89	(0.73, 1.08)	0.23
Flossed in the past 7 days[h]	1.44	(1.09, 1.89)	0.01	0.94	(0.82, 1.08)	0.37	0.94	(0.77, 1.15)	0.56	0.84	(0.70, 1.00)	0.05
Baseline covariates at Wave 3												
Sex												
Male	Ref.			Ref.			Ref.			Ref.		
Female	1.08	(0.85, 1.37)	0.51	1.08	(0.93, 1.24)	0.31	0.99	(0.81, 1.20)	0.88	0.89	(0.75, 1.05)	0.15
Age												

© 2022 Silveira ML et al. *JAMA Network Open.*

18-24	Ref.			Ref.			Ref.			Ref.		
25-34	**1.82**	**(1.31, 2.53)**	**<.001**	**0.72**	**(0.61, 0.84)**	**<.0001**	0.96	(0.72, 1.27)	0.75	**1.28**	**(1.06, 1.54)**	**0.01**
35-44	**1.89**	**(1.30, 2.74)**	**<.01**	**0.49**	**(0.41, 0.59)**	**<.0001**	1.20	(0.86, 1.67)	0.28	**1.38**	**(1.04, 1.84)**	**0.03**
45-54	**2.60**	**(1.77, 3.82)**	**<.0001**	**0.57**	**(0.48, 0.69)**	**<.0001**	**1.73**	**(1.27, 2.35)**	**<.001**	**1.90**	**(1.51, 2.40)**	**<.0001**
55-64	**2.18**	**(1.47, 3.22)**	**<.001**	**0.27**	**(0.21, 0.35)**	**<.0001**	**1.84**	**(1.28, 2.64)**	**<.01**	**2.36**	**(1.77, 3.15)**	**<.0001**
65+	**2.62**	**(1.63, 4.20)**	**<.001**	**0.17**	**(0.13, 0.24)**	**<.0001**	1.50	(0.99, 2.29)	0.06	**2.45**	**(1.73, 3.48)**	**<.0001**
Race/Ethnicity												
Non-Hispanic, White	Ref.			Ref.			Ref.			Ref.		
Non-Hispanic, Black	**1.62**	**(1.26, 2.09)**	**<.001**	0.96	(0.78, 1.17)	0.67	1.23	(0.92, 1.65)	0.16	**1.93**	**(1.56, 2.38)**	**<.0001**
Non-Hispanic other, including multi-racial	**1.75**	**(1.17, 2.61)**	**0.01**	1.08	(0.84, 1.38)	0.57	1.16	(0.74, 1.81)	0.51	**1.50**	**(1.09, 2.05)**	**0.01**
Hispanic	1.31	(0.94, 1.82)	0.11	1.06	(0.86, 1.31)	0.56	1.06	(0.80, 1.42)	0.68	**1.41**	**(1.09, 1.82)**	**0.01**
Educational attainment												
Less than high school or some high school, no diploma	Ref.			Ref.			Ref.			Ref.		
GED	1.24	(0.70, 2.21)	0.45	0.87	(0.63, 1.20)	0.39	1.00	(0.69, 1.44)	0.98	1.02	(0.72, 1.43)	0.92
High school graduate - diploma	1.34	(0.89, 2.01)	0.15	0.99	(0.77, 1.28)	0.95	0.80	(0.57, 1.14)	0.21	**0.70**	**(0.53, 0.93)**	**0.01**
Some college (no degree) or associate degree	1.42	(0.93, 2.17)	0.10	1.06	(0.86, 1.30)	0.59	**0.66**	**(0.49, 0.90)**	**0.01**	**0.50**	**(0.39, 0.65)**	**<.0001**
Bachelor's degree or more	1.16	(0.70, 1.92)	0.55	1.24	(0.95, 1.61)	0.12	**0.48**	**(0.32, 0.72)**	**<.001**	**0.40**	**(0.29, 0.57)**	**<.0001**
Annual household income												
Less than $25,000	Ref.			Ref.			Ref.			Ref.		
$25,000-$49,999	0.82	(0.63, 1.08)	0.17	0.89	(0.76, 1.04)	0.13	**0.68**	**(0.52, 0.88)**	**<.01**	**0.69**	**(0.55, 0.86)**	**<.01**
$50,000-$74,999	1.01	(0.70, 1.45)	0.97	0.94	(0.76, 1.17)	0.58	**0.49**	**(0.34, 0.72)**	**<.001**	**0.75**	**(0.60, 0.95)**	**0.02**
$75,000 or more	0.82	(0.57, 1.19)	0.29	0.83	(0.69, 1.02)	0.07	**0.30**	**(0.20, 0.45)**	**<.0001**	**0.41**	**(0.33, 0.52)**	**<.0001**

© 2022 Silveira ML et al. *JAMA Network Open.*

Cigarette pack-years												
0	Ref.			Ref.			Ref.			Ref.		
>0 to ≤5	**1.53**	**(1.16, 2.03)**	**<.01**	1.20	(1.00, 1.44)	0.05	**1.48**	**(1.07, 2.03)**	**0.02**	**1.34**	**(1.06, 1.70)**	**0.02**
>5 to ≤10	1.45	(0.97, 2.17)	0.07	1.23	(0.93, 1.63)	0.14	**1.79**	**(1.17, 2.74)**	**0.01**	1.13	(0.85, 1.52)	0.40
>10 to ≤15	**2.19**	**(1.36, 3.55)**	**<.01**	1.21	(0.84, 1.73)	0.30	1.62	(0.99, 2.64)	0.05	1.43	(0.96, 2.13)	0.08
>15 to ≤20	1.65	(1.01, 2.69)	0.05	**1.49**	**(1.07, 2.10)**	**0.02**	1.20	(0.75, 1.92)	0.44	1.51	(0.91, 2.49)	0.11
>20	**1.80**	**(1.24, 2.61)**	**<.01**	0.94	(0.69, 1.28)	0.67	**1.65**	**(1.05, 2.57)**	**0.03**	**1.44**	**(1.06, 1.96)**	**0.02**
Lifetime history of diabetes[h]	1.21	(0.91, 1.61)	0.19	1.16	(0.95, 1.43)	0.14	1.32	(0.98, 1.80)	0.07	**1.29**	**(1.03, 1.63)**	**0.03**

[a]Bone loss around teeth was defined based on a positive response to the question whether participants were told by a dentist, hygienist, or other health professional that they had lost bone around their teeth in the past 12 months regardless of missing data at any of the waves. Participants who either responded no to this question or were not asked this question because of not having seen a dentist in the past 12 months at Wave 4 and who either responded no this question or were not asked this question because of not having seen a dentist in the past 12 months or did not know or refused to report whether they have seen a dentist in the past 12 months at Wave 5 were categorized as not having the outcome.

[b]Bleeding after brushing/flossing was defined based on a positive response to the question whether participants had observed any bleeding after brushing or flossing, or due to other conditions in their mouth in the past 12 months regardless of missing data at any of the waves. Participants who responded no to this question at Waves 4 and 5 were categorized as not having the outcome.

[c]Loose teeth was defined based on a positive response to the question whether participants had any teeth become loose on their own, without an injury in the past 12 months regardless of missing data at any of the waves. Participants who responded no to this question at Wave 4 and who either responded no to this question or were not asked this question because of having had all their permanent teeth removed at Wave 5 were categorized as not having the outcome.

[d]One or more teeth removed was defined based on greater than zero teeth removed to the question regarding how many of participants' permanent teeth had been removed because of tooth decay or gum disease in the past 12 months regardless of missing data at any of the waves. Those who responded zero to this question at Waves 4 and 5 were categorized as not having the outcome.

[e]See supplemental table 1 for variable description.

[f]AHR: adjusted hazard ratio; 95% CI: 95% confidence intervals. Models are weighted using the Wave 5 all-waves weights for the Wave 1 Cohort.

[g]Current established use (yes/no) for cigarettes was defined as having smoked at least 100 cigarettes in one's lifetime and now smokes every day or some days. For other products, current established use (yes/no) was defined as ever using the product "fairly regularly" and now smokes/uses every day or some days. Ref: yes vs. no.

[h]Ref: yes vs. no.

© 2022 Silveira ML et al. *JAMA Network Open.*
