# Supplementary material for: Tobacco Use and Incidence of Adverse Oral Health Outcomes Among US Adults in the Population Assessment of Tobacco and Health Study
Source: JAMA Netw Open. 2022 Dec 9;5(12):e2245909. doi: 10.1001/jamanetworkopen.2022.45909 (PMC9856400; doi:10.1001/jamanetworkopen.2022.45909)
Supplement: Supplement 2. — Data Sharing Statement [file jamanetwopen-e2245909-s002.pdf]

Data Sharing Statement

Silveira. Tobacco Use and Incidence of Adverse Oral Health Outcomes Among US Adults in the Population Assessment of Tobacco and Health Study. *JAMA Netw Open*. Published December 09, 2022. doi:10.1001/jamanetworkopen.2022.45909

Data
Data available: Yes
Data types: Deidentified participant data
How to access data: https://doi.org/10.3886/Series606
When available: With publication

Supporting Documents
Document types: Informed consent form, Other (please specify)
Additional Information: User Guide, questionnaires, codebooks, non-response bias analysis reports
How to access documents: https://doi.org/10.3886/Series606
When available: With publication

Additional Information
Who can access the data: Anyone requesting the data via an application to the Restricted-use Files
Types of analyses: for any purposes
Mechanisms of data availability: after approval of a proposal and with a signed data access agreement
Any additional restrictions: None
